# *Physalis peruviana* calyces extract ameliorate oxidative stress, inflammation, and immune loss in rats-exposed to hexaflumuron

**DOI:** 10.1186/s12906-025-04750-z

**Published:** 2025-01-22

**Authors:** Mona M. Hashem, Eman I. Hassanen, Neven H. Hassan, Marwa A. Ibrahim, Marwa Y. Issa, Mohamed A. Farag, Sherif A. Hamdy

**Affiliations:** 1 Pharmacognosy Department, Faculty of Pharmacy, Cairo University, Kasr el Aini st, P.B. 11562, Cairo, Egypt; 2 Department of Pathology, Faculty of Veterinary Medicine, Cairo University, Giza, Egypt; 3 Department of Physiology, Faculty of Veterinary Medicine, Cairo University, Giza, Egypt; 4 Department of Biochemistry and Molecular Biology, Faculty of Veterinary Medicine, Cairo University, Giza, Egypt

**Keywords:** *Physalis peruviana*, Hexaflumuron, Immune system toxicity, Oxidative stress, Anti-inflammatory, UPLC-MS/MS analysis

## Abstract

**Background:**

Hexaflumuron (HFM), a common pesticide, can disrupt the immune system and cause oxidative stress. This study investigated the potential of *Physalis peruviana* L. calyces extract (PP) to counteract these effects in rats.

**Methods:**

Rats were divided into 6 groups including control, PP-treated, HFM-exposed, and co-treated (HFM + PP) groups. Immune function, antioxidant activity, and organ damage were assessed. Furthermore, UPLC-MS/MS analysis identified potential bioactive compounds in PP extract.

**Results:**

HFM exposure suppressed immune responses and caused organ damage. Notably, the co-administration of PP extract with HFM reversed these effects, indicating its ability to reduce oxidative stress and protect the immune system. UPLC-MS/MS analysis of PP calyces ethanolic extract revealed its richness in various health-promoting metabolites, including acyl sucrose sugar, withanolides, and flavonoids, which may provide valuable insight into the underlying mechanisms of PP’s calyces protective effects against HFM toxicity.

**Conclusions:**

This study provides novel insights into the potential of *P. peruviana* L. calyces ethanolic extract as a natural agent to counteract the harmful effects of HFM exposure. These findings have significant implications for developing effective strategies to mitigate pesticide-induced toxicity and promote human health.

**Supplementary Information:**

The online version contains supplementary material available at 10.1186/s12906-025-04750-z.

## Background

The widespread use of pesticides, including hexaflumuron (HFM), in veterinary and agricultural practices contributes significantly to environmental pollution due to residual contamination; the residues of these pesticides can be detected in food sources in significant quantities, posing health hazards such as neurological disorders, respiratory disorders, reproductive issues, and cancer risks [1]. Furthermore, several studies have shown that environmental pollutants, including heavy metals and pesticides, can induce toxicity in non-target organisms via mechanisms involving inflammation and oxidative stress [2]. Various categories of pesticides can instigate oxidative stress, resulting in tissue damage through processes such as free radicals generation, modifications in antioxidant defenses, and lipid peroxidation [3].

Pesticide-induced oxidative stress has emerged as a key area of toxicological studies over the past decade due to its potential implications for immunotoxicity [4]. Exposure to pesticides can lead to diverse immune system dysfunctions, including impaired neutrophil and macrophage activity, decreased thymocyte numbers, altered mitogen-induced cell proliferation, reduced antibody-dependent cellular cytotoxicity, and dysregulated cytokine secretion [5]. Additionally, pesticide exposure may contribute to the development of autoimmune diseases, immunosuppression, and hypersensitivity reactions [6].

*Physalis peruviana* L. (Solanaceae), a member of the *Physalis* genus, boasts a diverse range of vernacular names, including cape gooseberry, poha, goldenberry, husk tomato, tomatillo, alkekengi, and ground cherry. Notably, it is also known as Harankash in Egypt, where it is popular as a local snack food [7]. *Physalis peruviana* L. calyces, the protective husks surrounding the cape gooseberry fruit, have attracted increasing attention due to their potential therapeutic benefits.

Several studies have been reported on the immunomodulatory capabilities of *P. peruviana* L. (cape gooseberry), proposing its capacity to impact the functionality of the immune system across the examination of immune cell behavior, the production of different types of cytokines (a key immune signaling molecule), and reduction of oxidative stress. These investigations encompass a variety of methodologies (in-vivo and in-vitro studies) to prove these effects. A study by Mier-Giraldo et al. reported the immunomodulatory properties of (PP) ripe fruit extract. The extract exhibited dose-dependent suppression of interleukin-6 (IL-6), interleukin-8 (IL-8), and monocyte chemoattractant protein-1 (MCP-1) expression in both human cervical cancer (HeLa) and murine fibroblast (L929) cells [8]. Sang-Ngern et al. revealed that withanolides from aerial parts of (PP) have potent nitric oxide inhibitory activity in LPS-activated RAW 264.7 cells, and significantly inhibited the TNF-*α*-induced NF-*k*B activity [9]. In vitro investigation by Martínez et al. unveiled the immunomodulatory potential of (PP) calyces extract, showcasing its ability to exhibit anti-inflammatory characteristics through suppressing proinflammatory cytokine production in macrophages [10]. Moreover, (PP) sucrose esters rich fraction demonstrated significant anti-inflammatory activity against TBS-induced colitis via inhibition of proinflammatory mediators (INOS, Cox-2), and cytokines (TNF-α, IL-6, IL-1β), in addition to notable ability to regenerate epithelial tissue associated with a concurrent increment of goblet cells count, as well as MUC-2 and TFF-3 gene expression levels [11]. Franco et al., showed the significant anti-inflammatory activity of sucrose esters from (PP) calyces in an animal model and the in-vitro effect on NO., PGE2, and TNF-α production from LPS-stimulated murine macrophages [12]. Several studies examined the potential of *P. angulata* calyces to treat inflammatory bowel disease. *P. angulata* Extract reduced inflammation in immune cells (macrophages) in-vitro studies and lessened disease symptoms in mice with colitis. *P. angulata* may regulate immune cells to improve gut health, offering promise as a future treatment for inflammatory bowel disease [13]. Furthermore, comprehensive pharmacological investigations have delineated various mechanisms of action within the oxidative damage pathways of *P. peruviana*. The study conducted by Toro et al.. highlighted the notable in vitro scavenging potential of (PP) calyces’ ethanol extract against superoxide and nitric oxide radicals [14]. Al-Olayan et al., revealed that (PP) extract exhibited a concentration-dependent antiradical activity resulting from a reduction of DPPH, superoxide anion, TBARS, and nitric oxide radicals to non-radical form [15].

Phytochemical studies in *P. peruviana* L. calyces revealed a diverse range of secondary metabolites, including flavonoids, with rutin (quercetin 3-*O*-rutinoside) being the most abundant flavonoid [16]. Terpenoid glycosides and steroidal lactones, particularly withanolides, have been identified [12, 17–19]. Other compounds identified in *P. peruviana* calyces include physalins, oxylipins, and sucrose esters [9, 20, 21].

Given the extensive utilization of pesticides, exposure to these substances occurs daily for all individuals, regardless of direct involvement in their handling. A considerable quantity of hexaflumuron as a pesticide is released into the environment during application processes, with a substantial portion affecting non-target species and the surrounding ecosystem. Despite the existence of risk assessment information before its introduction into the market, continued evaluation of the potential risks posed by hexaflumuron to non-target organisms is imperative, especially considering its prolonged use over the years. Despite the previously reported activities of *P. peruviana*, the immunomodulatory properties of *P. peruviana* calyces remain largely unexplored. This study aims to address this knowledge gap by investigating the calyces’ potential to modulate the immune system in the context of inflammation and oxidative stress induced by HFM in a rat model. Furthermore, the study identifies the potential bioactive phytochemicals within the calyces that may contribute to the observed immunomodulatory effects.

## Materials and methods

### Chemicals and solvent

Hexaflumuron pure 99%, 1-[3,5-dichloro-4-(1,1,2,2-tetrafluoroethoxy) phenyl]-3-(2,6-difluorobenzoyl) urea with a chemical formula C_16_H_8_Cl_2_F_6_N_2_O_3_ was obtained from Kafr El-Zayat Pesticides & Chemical Company (Kafr El-Zayat, Garbaia, Egypt). It is freshly prepared in deionized distilled water according to the required concentration. All chemicals in this study were of analytical grade. HPLC grade solvents-acetonitrile and formic acid for LC-MS were obtained from Sigma-Aldrich (St. Louis, USA).

### Plant material collection and authentication

The calyces of *P. peruviana* L. were collected in February-March 2023 from fruit traders in the local market, Cairo, Egypt. The plant was identified at the Agricultural Research Center, Giza, Egypt, and authenticated by Eng. Threase Labib, consultant in Orman Garden and National Gene Bank, Ministry of Agriculture. A voucher specimen (No. 8.4.23) is deposited in the Herbarium of the Department of Pharmacognosy, Faculty of Pharmacy, Cairo University. The plant name was verified with the plant list http://www.theplantlist.org on the 4th of July 2023. Calyces were manually separated from the mature fruit, washed with distilled water, and dried at room temperature for 72 h in the darkness. The air-dried sample (2 kg) was ground into powder form, then vacuum-sealed and stored at -20 °C until further assays.

### Preparation of the ethanolic extract for biological study

Freez-dried powdered cape gooseberry calyces (2 Kg) were macerated in 5 L ethanol (70%) for three days, then filtered and combined with being evaporated by rotary vapor at a temperature not exceeding 55 ^0^C to yield dark brown amorphous residue (65 g), the percentage yield of extract was at 3.25%. The residue was stored in the refrigerator at 4 ^0^C for biological analyses.

### Sample Preparation for UPLC-MS analysis

Two grams of freeze-dried cape gooseberry calyces were homogenized with 5 mL of 100% methanol containing 10 µg/mL of umbelliferon as an internal standard for LC-MS quantification. A high-shear homogenizer (e.g., Turrax mixer) operating at 11,000 rpm for five 20-second intervals facilitated efficient cell disruption. The homogenate was then subjected to vigorous vortexing and centrifugation (12,000 g, 5 min) to separate the extracted metabolites from the plant debris. The supernatant was subsequently passed through a 22 μm filter for further purification. An aliquot of 500 µL was loaded onto a preconditioned C-18 solid-phase extraction (SPE) cartridge (500 mg bed volume) equilibrated with methanol and water. Elution was achieved sequentially with 3 mL of 70% methanol and 3 mL of 100% methanol. The combined eluent was then evaporated to dryness under a stream of nitrogen. The resulting residue was finally reconstituted in 500 µL of methanol for subsequent UPLC-MS analysis.

### High-resolution UPLC-MS analysis

The UPLC-ESI-qTOF-MS analysis employed in this study followed the methodology established by Farag et al. 2021 [22].

### Animals and experimental design

Forty-two male albino Wistar rats (150 ± 20 g) were obtained from the Department of Veterinary Hygiene and Management’s Animal House, Faculty of Veterinary Medicine, Cairo University, Egypt. Animals were reared in plastic cages, fed with standard commercial pelleted feed, and water was supplied *ad libitum*. They were inspected for health status and acclimatized to the research laboratory environment for two weeks before use. This study is performed in accordance with ARRIVE guidelines (https://arriveguidelines.org). The institutional animal care permitted all the procedures and experimental designs and use committee (IACUC) of Cairo University (Approval number: Vet CU12102021361).

Rats were randomly divided into six groups (*n* = 7) and given the following materials daily by oral gavage for 28 days. Group 1 received normal saline and was kept as a negative control group. Groups 2 and 3 received *P. peruviana* calyces extract at 200 and 400 mg/kg b.wt., respectively [23]. Group 4 received hexaflumuron (HFM) at 11 mg/kg bwt., corresponding to 1/10 LD_50_. Groups 5 and 6 co-administered HFM with *P. peruviana* calyces extract with the same doses as mentioned above, two hours in between. The dose of HFM was selected based on its LD_50_, which was reported to be 110 mg/kg bwt [24, 25]. Throughout the experimental period, rats in all groups were monitored daily for any clinical symptoms or mortality and weighed weekly until the end of the study.

### Sampling

After 28 days, rats were anesthetized with intramuscular injections of xylazine (10 mg/kg) and ketamine (90 mg/kg) to collect blood samples from the orbital sinus. Some blood samples were collected in EDTA-containing tubes, whereas others were centrifuged at 3500 rpm for 5 min to obtain clear serum samples preserved at -20 °C till used for biochemical analysis. After that, rats were euthanized by (humane killing) using a CO_2_ chamber to collect spleen and thymus samples. Part of these samples was preserved at − 80 °C till used for biochemical evaluations and molecular studies. In contrast, the other part was fixed in 10% neutral buffered formalin to perform histopathological and immunohistochemical examinations.

### Total and Differential Leucocytic Count

Total leucocytic count (TLC) was performed according to Dacie and Lewis’s methods [26]. Freshly collected blood samples of 20 µL were spread on clean slides as a thin film. Each smear was left to air dry, fixed with methanol for 2–3 min, and then labeled. After that, blood smears were stained with 10% Giemsa’s stain (Aldrich), examined under light microscopy, and the differential leucocytic count was performed according to Schalm 2022 [27].

### Immunoglobulin (IgG and IgM) level assay

Total IgG and IgM levels were measured using commercial ELISA kits for rats, The IgG level was measured with a rat IgG ELISA kit (ab189578#; Abcam, Cambridge, UK), and IgM concentration was measured using Abcam rat IgM ELISA kits (ab157738#; Cambridge, MA) according to the manufacturer’s instruction.

### Oxidative stress marker assays

Serum Malondialdehyde (MDA) was assessed through spectrophotometric techniques, as previously detailed [28]. The determination of serum total antioxidant capacity (TAC) followed the methodology outlined by Koracevic et al. 2001 [29]. The CAT (catalase assay) activity was evaluated per the procedure Alam et al., described [30].

### RT-PCR quantification of mRNA levels of IL-1, IL-6, IL-10, Cox-2 and TGF-1b genes

The total RNA of the spleen tissues was isolated using RNeasy Mini Kit (Qiagen Cat No./ID: 74104). The Super Script Reverse Transcriptase (Thermo-Scientific) was used for the synthesis of first-strand cDNA according to the manufacturer’s instructions. Quantitative PCR was performed using SYBR™ Green PCR Master Mix (Thermoscientific Cat number: 4309155). ABI Prism Step-OnePlus Real-Time PCR System (Applied Biosystems) according to the manufacturer’s instructions. The primer sets of the assessed genes are listed in Table 1. The ACTB was used as an internal control to normalize the relative m-RNA expression level.

**Table 1:** The primer sets of the studied genes

Genes	Sense	Antisense	Amplicon	Accession no
Il-1β	TTGAGTCTGCACAGTTCCCC	GTCCTGGGGAAGGCATTAGG	161	NM_031512.2
Il-6	CCAGTTGCCTTCTTGGGACT	TCTGACAGTGCATCATCGCT	224	NM_012589.2
Il-10	TCCCTGGGAGAGAAGCTGAA	CCTGCAGTCCAGTAGATGCC	234	NM_012854.2
TGF-1β	TACGCCAAAGAAGTCACCCG	GTGAGCACTGAAGCGAAAGC	357	NM_021578.2
Cox-2	AGGAGCATCCTGAGTGGGAT	AGAAGCGTTTGCGGTACTCA	459	L25925.1
ACTB	CCGCGAGTACAACCTTCTTG	CAGTTGGTGACAATGCCGTG	297	NM_031144.3

**Abbreviation**: *IL-1b*, interleukin 1 beta; *IL-6*, interleukin 6; *IL-10*, interleukin 10; TGF-1*b*, transforming growth factor-1 beta; Cox-2, cyclooxygenase 2; ACTB, actin biotin housekeeping gene

### Histopathological examination

Formalin-fixed spleen and thymus tissue specimens were dehydrated using the ascending grade of ethanol, purified by xylene, embedded in paraffin wax, and sliced at 4.5 μm to obtain paraffin-embedded tissue sections stained by H&E and examined under light Olympus microscope to determine any pathological alterations [31].

All the observable pathological parameters were graded using a classical semiquantitative scoring system to assess the degree of lymphocytolysis between different groups. The five-pointed ordinal scale was used as the following: (0) none [1], mild < 25% [2], moderate 25%:50% [3], severe 50%:75%, and [4] extensive severe > 75% tissue damage [32]. Additionally, Image J software was used to measure the diameter of lymphoid follicles, the thickness of the marginal zone, the thickness of the periarteriolar sheath, and the thickness of the thymic cortex based on the methods portrayed by [33].

### Immunohistochemical studies

Immunohistochemical analysis was undertaken to ascertain the protein expression of caspase-3 (a marker for apoptosis), CD4 (a marker for T-helper cells), and CD8 (a marker for T-cytotoxic cells) within the spleen tissue sections utilizing avidin-biotin-peroxidase complex (ABC). Following deparaffinization, the tissue sections were subjected to incubation with distinct primary antibodies sourced from Abcam Ltd., USA, and subsequently treated with the necessary reagents for the ABC reaction via the Vectastain ABC-HRP Kit provided by Vector Laboratories. Post-incubation, slides were designated with peroxidase and stained utilizing the DAB-chromogen substrate from Sigma, and after that examined through a light Olympus microscope. The quantification of the mean percentage of various immunostaining expressions across diverse groups was conducted using Image J software.

## Results

### UPLC-Orbitrap HRMS metabolites profiling of *Physalis peruviana* calyces

**Fig. 1:**
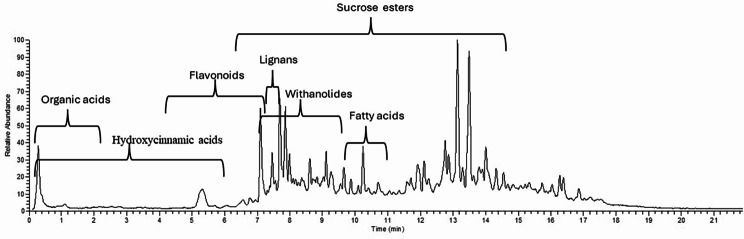
UPLC-Orbitrap HRMS total ion current (TIC) chromatogram of *Physalis peruviana* L. calyces extract in the negative ESI mode

UPLC-QTOF-MS analysis (Fig. 1) revealed the presence of various secondary metabolites in the cape gooseberry calyces. Sucrose esters amounted to the most abundant class. Other major metabolite classes included withanolides, organic acids, phenolics, flavonoids, lignans, and fatty acids. The identified metabolites are listed in Table 2.

**Table 2:** Metabolite identification in *Physalis peruviana* L. calyces methanolic extract via UPLC-Orbitrap HRMS in the negative ESI mode

Peak no.	Rt (min)	M-H^−^	Molecular Ion Formula	Error (ppm)	MS^*n*^	Identification
Organic acids						
O1	0.30	133.01434	C_4_H_5_O_5_	6.51	191, 115, 111, 105, 97, 89, 87, 75, 71, 59	Malic acid
O2	0.40	117.01958	C_4_H_5_O_4_	4.12	99, 89, 73, 59	Succinic acid
O3	0.85	191.01981	C_6_H_7_O_7_	6.18	173, 146, 111, 101, 85, 59	(Iso)citric acid
O4	0.95	153.01942	C_7_H_5_O_4_	7.74	138, 125, 109, 89, 79	Protocatechuic acid
O5	2.28	121.02950	C_7_H_5_O_2_	9.04	95, 78	Benzoic acid
Hydroxycinnamic acids						
H1	0.06	163.0402	C_9_H_7_O_3_	1.2	144, 135, 119, 101, 90, 85, 81,75, 59	*p*-Coumaric acid
H2	1.02	353.08707	C_16_H_17_O_9_	1.02	191, 179, 161,134	3-*O*-Caffeoylquinic acid
H3	1.68	353.08707	C_16_H_17_O_9_	1.36	309, 285, 263,191,179,173,155,135,111	4-*O*-Caffeoylquinic acid
H4	2.32	179.03468	C_9_H_7_O_4_	5.55	164, 151, 149, 141,135, 117,109	Caffeic acid
H5	2.90	367.10281	C_17_H_19_O_9_	0.494	349, 331, 321,193, 173, 134	3-*O*-Feruoylquinic acid
H6	3.23	325.09158	C_15_H_17_O_8_	5.16	307, 279, 265, 234, 205,187,163,145	*p*-Coumaric acid-*O*-hexoside
H7	3.42	337.09235	C_16_H_17_O_8_	2.45	319, 301, 293,191, 173, 163	3-*p*-Coumaroylquinic acid
H8	4.55	385.11398	C_17_H_21_O_10_	0.770	367, 339, 235, 317, 295, 265, 247, 223, 205, 193,179,161,149,135,119	Sinapoyl-*O*-hexoside
H9	4.88	355.10324	C_16_H_19_O_9_	1.102	338, 295, 265, 235, 217,193,191,175,160,149,134	Ferulic acid-*O*-hexoside
H10	6.48	471.1414			323, 309, 179, 161,	Sibirioside A
Phenolics & Flavonoids						
F1	0.31	289.06985	C_15_ H_13_ O_6_	-2.81	281, 271, 253, 243, 191,125	(Epi)catechin
F2	4.30	771.19690	C_33_H_39_O_21_	1.2	609, 463, 301	Quercetin-*O*-hexosyl-rhamnosyl hexoside
F3	5.00	609.29120	C_27_H_29_O_16_	0.78	606, 577, 563, 463, 301, 271, 255, 228,179	Quercetin-3*-O-*rutinoside
F4	7.16	463.08859	C_21_H_19_O_12_	0.84	445, 417, 404, 372, 342, 325, 313, 301, 271, 255, 217,179	Quercetin-3-*O*-hexoside
F5	7.36	593.15179	C_27_H_29_O_15_	1.06	547, 447, 326, 285, 284, 257, 229, 210,187, 173	Kaempferol-3*-O-*rutinoside
F6	7.46	447.09329	C_21_H_19_O_11_	0.002	429, 401, 357, 343, 327, 301, 285, 255, 243, 227,179	Kaempferol-3*-O-*hexoside
F7	5.00	609.15	C_27_H_29_O_16_	0.78	606, 577, 563, 463, 301, 271, 255, 228,179	Quercetin-3*-O-*rutinoside (rutin)
Lignans						
L1	7.19	521.20306	C_30_H_41_O_8_	0.43	503, 475, 463, 449, 431, 415, 363, 359, 341, 329, 311, 298, 281, 259, 178	Lariciresinol-*O*-hexoside
L2	7.20	359.15036	C_20_H_23_O_6_	0.97	341, 329, 311, 283, 255, 241, 223, 217, 205,187, 171,161,115	Lariciresinol
L3	7.17	557.24561	C_30_H_39_O_9_	0.92	539, 521, 497, 480, 455, 425, 395, 359, 341, 313, 263	Dihydroxy lariciresinol-hexoside
Withanolides						
W1	6.95	535.25421	C_28_H_39_O_10_	0.815	517, 489, 413, 373, 357, 313, 303, 285	Withangulatin G
W2	7.33	519.26007	C_28_H_39_O_9_	-1.3	501, 483, 473, 456, 379, 361,357, 343, 317, 313	2,3-Dihydro-27-hydroxy-4β-hydroxywithanolide E
W3	7.49	501.2494	C_28_H_37_O_8_	5.3	483, 457, 439, 421, 359, 315, 297	17,27-Dihydroxylatedwithanolide D
W4	7.54	519.2601	C_28_H_39_O_9_	0.93	507, 501, 477, 463, 377, 359, 357, 341, 315	24,25-Dihydro-4,27-dihydroxylatedwithanolide E
W5	9.21	487.2701	C_28_H_39_O_7_	1.09	469, 451, 443, 425, 407, 389, 361, 343, 345, 327, 299, 283, 271, 243, 187,141	2,3-Dihydro-27-hydroxylated withanolide D
W6	9.23	485.2550	C_28_H_37_O_7_	0.83	469, 451, 443, 425, 407, 389, 361, 345, 327, 299, 283, 271, 243,187,141	Withanolide E
W7	9.35	469.2607	C_28_H_37_O_6_	0.55	451,439, 425, 407, 381, 327, 345, 309, 283, 265, 313, 185,141	Withanolide D
Sucrose Esters						
S1	0.61	411.16013	C_16_H_28_O_12_	2.45	341, 323, 295, 243	*O*-Isobutanoylsucrose
S2	2.27	481.11182	C_20_H_33_O_13_	0.75	463, 445, 435, 393, 359, 353, 323, 305, 221, 179	Di-*O*-isobutanoylsucrose
S3	6.51	535.23120	C_24_H_39_O_13_	-13.6	517, 491, 459, 419, 398, 393, 359, 323, 281, 236	*O*-Isobutanoyl-O-octenoylsucrose
S4	10.72	565.24634	C_26_H_45_O_13_	-4.85	495, 477, 449, 411, 393, 341, 323, 305, 249, 231	*O*-Decanoyl-*O*-isobutanoylsucrose
S5	10.75	607.23590	C_28_H_47_O_14_	2.8	593, 537, 509, 481, 463, 431, 393, 323, 305	Di-*O*-isobutanoyl-*O*-octanoylsucrose
S6	11.75	647.30121	C_31_H_52_O_14_	-1.01	577, 547, 539, 519, 493, 475, 423, 411, 393, 375, 341, 323, 305, 243	*O*-Decanoyl-*O*-isobutanoyl-*O*-(2 methylbutenoyl)sucrose
S7	11.96	635.32770	C_30_H_51_O_14_	0.57	565, 547, 481, 393	*O*-Decanoyl-di-*O*-isobutanoylsucrose
S8	12.02	649.34222	C_31_H_53_O_14_	-1.17	565, 495, 477, 433, 411, 393, 341, 243, 323	*O*-Decanoyl-O-isobutanoyl-*O*-(2-methylbutanoyl)sucrose
S9	12.37	747.33679	C_37_H_64_O_15_	0.3	733, 677, 663, 575, 565, 551, 491, 481, 463, 411, 393, 323,	Di-*O*-isobutanoyl-*O*-dodecanoyl-*O*-(2-methylbutanoyl)sucrose
S10	12.76	763.3724	C_35_H_57_O_15_	2.15	717, 563, 481, 411, 342	*O*-Decanoyl-*O*-diisobutanoyl-*O*-(2-methylbutenoyl)sucrose
S11	13.14	705.37152	C_34_H_57_O_15_	3.29	653, 617, 551, 481, 463	Peruviose A
S12	13.28	691.36713	C_33_H_55_O_15_	-0.4	621, 565, 551, 537, 519, 477, 449, 411, 393, 323, 279	*O*-Nonanoyl- tri-*O*-isobutanoylsucrose
S13	13.29	677.35101	C_32_H_53_O_15_	0.6	705, 687, 677, 607, 593, 537, 495, 469, 425, 411, 393, 342	*O*-Octanoyl-tri-*O*-isobutanoyl-sucrose
S14	13.52	719.38470	C_35_H_59_O_15_	-0.2	701, 635, 565, 547, 481, 463	Peruviose B
S15	14.13	733.41589	C_37_H_66_O_14_	1.42	715, 689, 663, 645, 579, 561, 535, 491, 481, 473, 463, 393, 351, 323, 265	*O*-Dodecanoyl-*O*-isobutanoyl-*O*-nonanoylsucrose
Fatty acids						
A1	9.7	301.2019	C_16_H_30_O_5_	3.3	283, 241, 239, 2 27	Hydroxyhexadecandioic acid
A2	9.77	325.2020	C_18_H_29_O_5_	3.2	307, 289, 281, 201, 171	Trihydroxy-octadecatrienoic acid
A3	9.78	329.23346	C_18_H_33_O_5_	2.5	309, 291, 283, 213, 201, 171	Trihydroxy-octadecenoic acid
A4	10.72	313.23819	C_18_H_33_O_4_	2.7	295, 283, 255	Dihydroxy-octadecenoic acid

### Total and Differential Leucocytic Count

Extract subjected to chemical profiling was further subjected to bioassays to assess its mitigation action against oxidative stress, inflammation, and immune loss decline in rats exposed to HFM. Data from Table 3 revealed a statistically significant decrease in total leukocyte count (TLC) (leucopenia) and lymphocytes % (lymphocytopenia), along with a marked increase in neutrophils % in the HFM-intoxicated group compared to the control group. Conversely, treatment with either dose of *P. peruviana* calyces extract resulted in a dose-dependent improvement in TLC, lymphocyte percentage, and neutrophil percentage compared to the untreated HFM group. Notably, no significant differences were observed between groups regarding eosinophil and monocyte percentages.

**Table 3:** Effect of HFM and/or PP calyces extract on total and differential leukocytic count

	Control	PP Low dose	PP High dose	HFM	HFM + PP Low dose	HFM + PP High dose
TLC (10^3^/mm^3^)	7.33 ± 0.82	7.50 ± 0.54	7.17 ± 0.31	3.83 ± 0.80 *	6.67 ± 0.67 ^	7.00 ± 0.89 ^
Neutrophil %	19.67 ± 2.60	19.83 ± 1.81	22.00 ± 2.63	35.33 ± 2.39 *	24.50 ± 1.87 ^	19.30 ± 2.15 ^
Lymphocyte %	77.50 ± 2.36	77.33 ± 1.93	75.17 ± 2.47	60.50 ± 2.82 *	71.33 ± 2.18 ^	78.16 ± 2.15 ^
Eosinophil %	1.83 ± 0.31	1.17 ± 0.16	1.50 ± 0.22	1.17 ± 0.17	2.00 ± 0.26	1.33 ± 0.21
Monocyte %	1.50 ± 0.22	1.00 ± 0.26	1.18 ± 0.31	1.50 ± 0.43	2.16 ± 0.40	1.16 ± 0.31

Values are presented as mean ± SE (*n* = 6–7). (*) means significant difference VS control. (^) means significant difference VS HFM at (*P* < 0.05)

### Immunoglobulin (IgG and IgM) levels assay

Table 4 shows a notable decline in both IgM and IgG levels following exposure to HFM compared to the control group. In contrast, administration of *P. peruviana* calyces extract at either high or low doses demonstrated a significant increase in IgM and IgG levels in a dose-dependent manner compared to the group exposed solely to HFM.

**Table 4:** Effect of HFM and/or PP calyces extract on IgM and IgG levels

	Control	PP Low dose	PP High dose	HFM	HFM + PP Low dose	HFM + PP High dose
IgM (µg/ml)	3.12 ± 0.19	3.52± 0.26	3.31 ± 0.27	1.44 ± 0.02*	2.27 ± 0.11^*	2.65 ± 0.13^
IgG(µg/ml)	29.21 ± 2.00	24.62 ± 1.1	25.26 ± 0.80	17.27 ± 0.3 *	23.9 ± 0.79^*	28.56 ± 0.78 ^

Values are presented as mean ± SE (*n* = 6–7). (*) means significant difference VS control. (^) means significant difference VS HFM at (*P* < 0.05)

### Oxidative stress assay

The data depicted in Table 5 indicated a significant increase in serum lipid peroxide (MDA) levels in the HFM group, along with a notable decrease in serum total antioxidant capacity (TAC) level and catalase (CAT) enzyme activity compared to the control group. Treatment with high and low doses of *P. peruviana* calyces extract resulted in a substantial rise in serum TAC and CAT activity, and a significant decrease in serum MDA levels when contrasted with the untreated HFM-exposed group.

**Table 5:** Effect of HFM and/or PP calyces extract on some oxidative stress markers

	Control	PP Low dose	PP High dose	HFM	HFM + PP Low dose	HFM + PP High dose
MDA (nmol/l)	21.42 ± 0.89	20.50± 0.76	23.83 ± 1.19	41.83 ± 1.58 *	32.66 ± 1.15^*	25.75 ± 0.66 ^
TAC (Mm/L)	1.85 ± 0.07	1.85 ± 0.56	1.83 ± 0.80	0.53 ± 0.06 *	1.04 ± 0.14 ^*	1.61 ± 0.16 ^
CAT(U/L)	19.17 ± 1.54	20.50 ± 1.84	20.83 ± 1.45	9.08 ± 0.91*	12.00 ± 1.16 ^*	19.50 ± 2.47 ^

Values are presented as mean ± SE (*n* = 21 microscopic fields/group). (*) significant difference Vs control group, (✓) significant difference Vs HFM group at (*P* < 0.05)

### RT-PCR quantification of mRNA levels of IL-1β, IL-6, IL-10, Cox-2 and TGF-1β

**Fig. 2:**
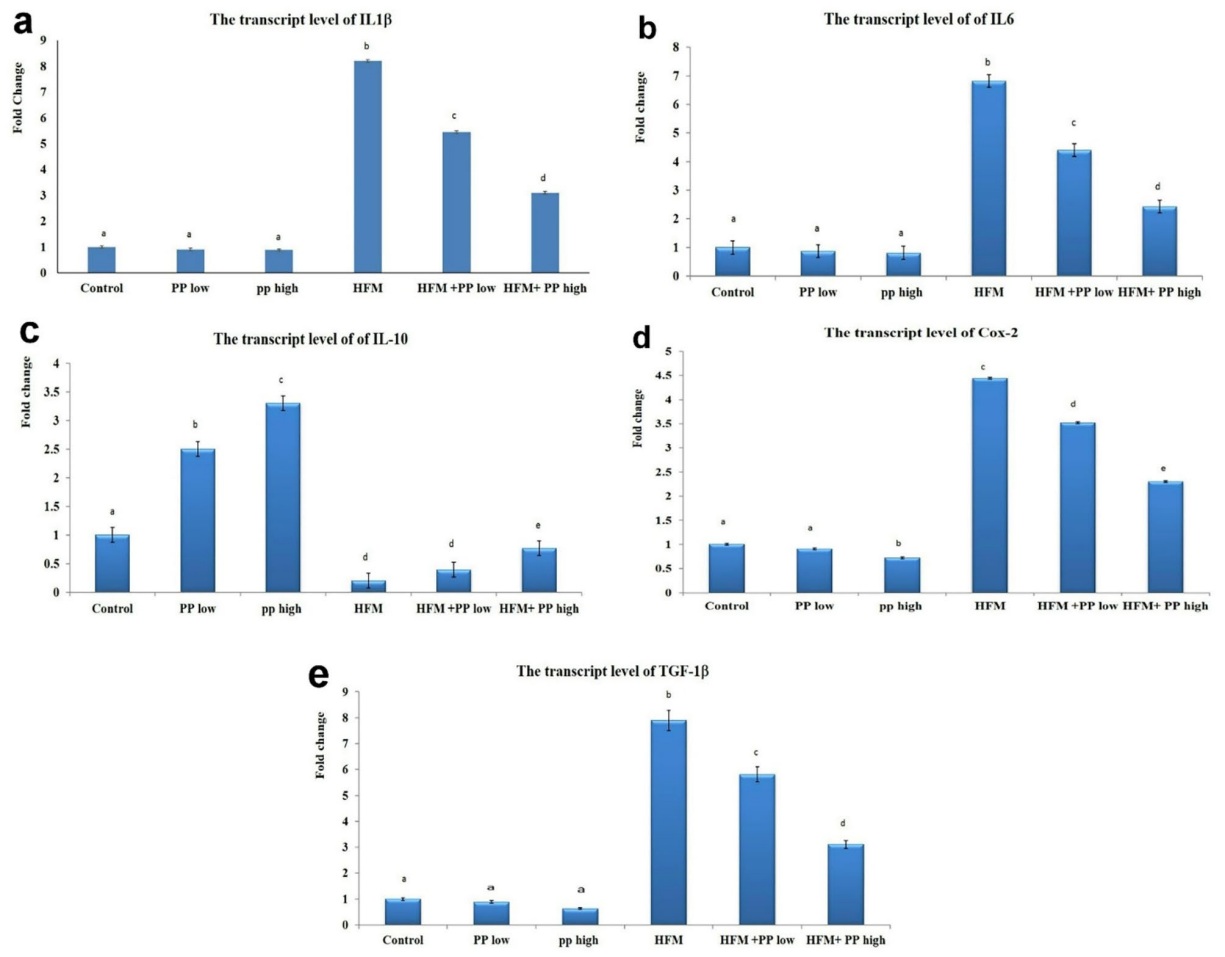
Bar charts representing the transcript levels of IL-1β (**a**), IL-6 (**b**), IL-10 (**c**), Cox-2 (**d**), and TGF-1β (**e**) genes across groups. Values are presented as mean ± SE (*n* = 6). Means with different superscript letters are significantly different at (*P* < 0.05). Bar charts representing the transcript levels of IL-1β (**a**), IL-6 (**b**), IL-10 (**c**), Cox-2 (**d**), and TGF-1β (**e**) genes across groups. Values are presented as mean ± SE (*n* = 6). Means with different superscript letters are significantly different at (*P* < 0.05)

The group exposed to HFM exhibited a noteworthy increase in mRNA levels of IL-1β, IL-6, Cox-2, and TGF-1β genes, accompanied by a decrease in IL 10 levels. In contrast to the control group. When compared to the HFM group, both groups receiving *P. peruviana* calyces extract at either low or high dosage levels demonstrated a decrease in the mRNA levels of IL1β, IL6, Cox-2, and TGF-1β genes, along with an increase in IL 10 levels (Fig. 2).

### Histopathological examination

**Fig. 3:**
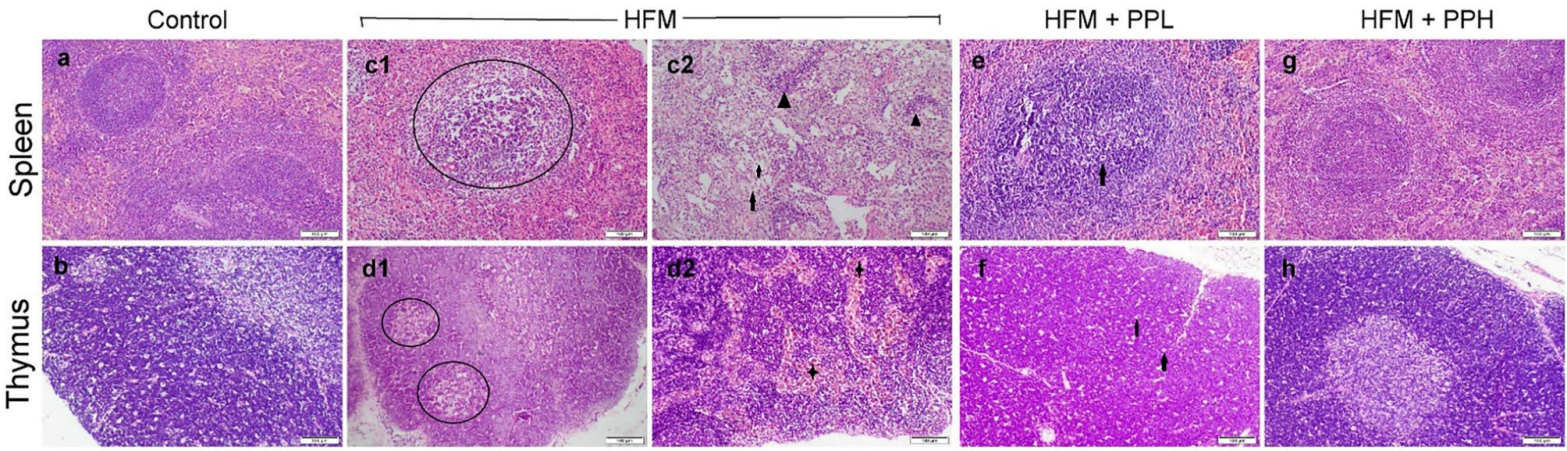
Histopathological examination of spleen and thymus obtained from various experimental groups. (**a, b**) control group showed normal microscopic picture of spleen and thymus. (**c, d**) HFM group showed severe lymphocytolysis (circle), atrophy of PALS (triangle), vacuolation of the splenocytes (arrow), hemorrhage of thymic medulla (star). (**e, f**) HFM + PPL (low dose) showed mild lymphoid depletion with aggregation of tangible macrophages (arrow). (**g, h**) HFM + PPH (high dose) showed normal histology of splenic lymphoid follicles in white pulp and thick thymic cortex

To confirm the biomarker assay results of immune modulation effects observed in the different treatments, histopathological examination of the spleen and thymus, which are the main sites of immune functions, was performed. All spleen and thymic sections obtained from the control groups displayed normal histological structure (Fig. 3a-b). Likewise, *P. peruviana* calyces extract receiving groups either at low or high doses, showed normal histological appearance of both spleen and thymus. On the other hand, the HFM receiving group showed marked lymphoid depletion and lymphocytolysis in both bursal-associated and periarteriolar lymphoid follicles of the splenic white pulp (Fig. 3c1). Moreover, the red pulp showed extensive vacuolation of the splenocytes (Fig. 3c2) along with hemorrhage and fibrin depositions. Regarding the thymus, there was a decrease in the thickness of the thymic cortex and multifocal areas of lymphocytic necrosis (Fig. 3d1). Some sections showed profuse hemorrhage in both cortex and medulla (Fig. 3d2). Otherwise, PP co-treated groups showed marked improvement in dose-dependent manner. The group receiving the low dose of PP along with HFM exhibited mild to moderate lymphoid depletion with the presence of a small number of foam cells (Fig. 3e). The thymus showed mild lymphocytosis in its cortex (Fig. 3f). Furthermore, the group receiving the high dose of PP along with HFM showed normal histological organization of both spleen and thymus sections and returned similar to those of the control group (Fig. 3g-h).

**Fig. 4:**
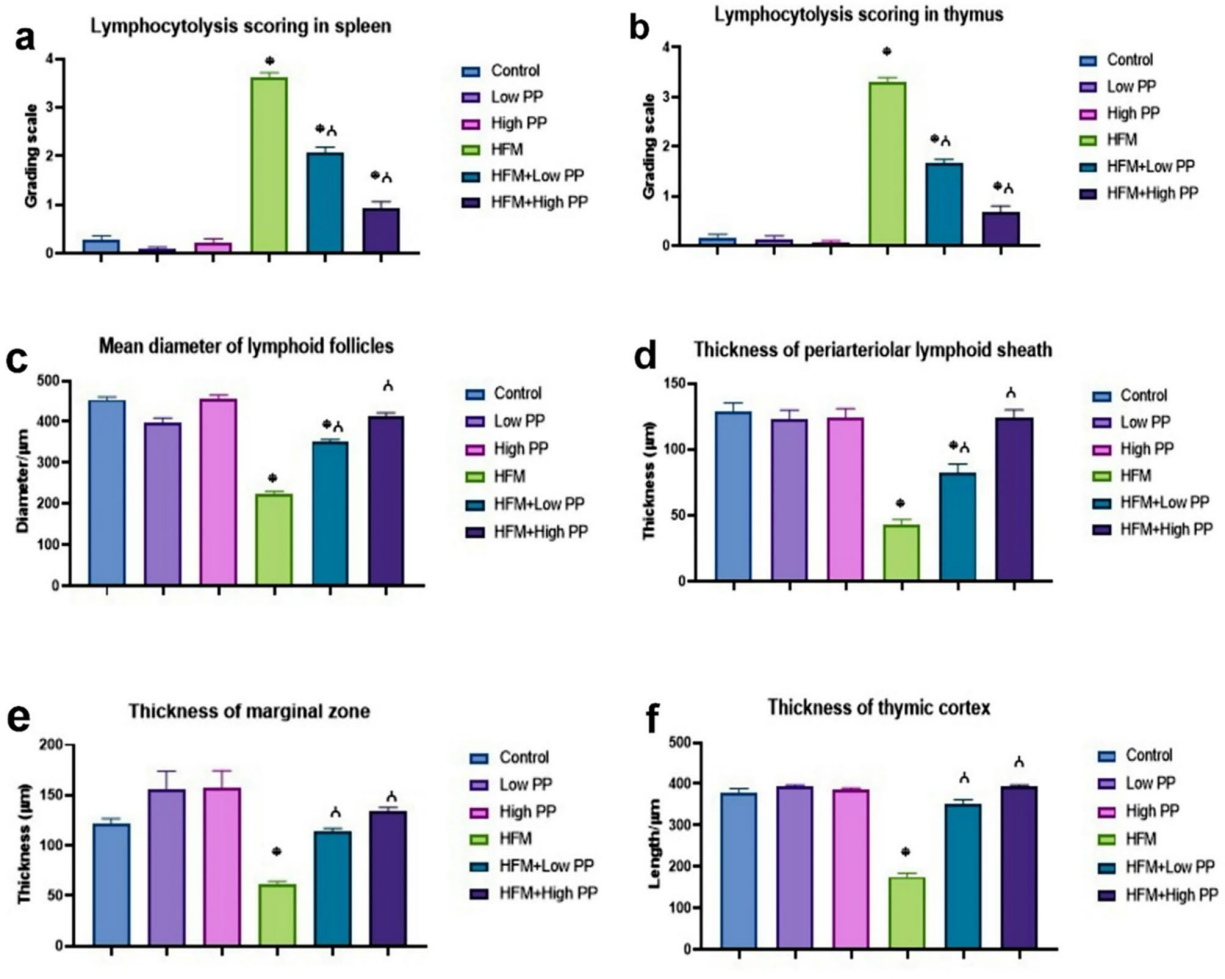
Bar charts representing semiquantitative scoring of both immune organs and histomorphometric analysis. (**a, b**) lymphocytolysis scoring in both spleen and thymus, respectively. (**c**) mean diameter of lymphoid follicles, (**d**) thickness of periarteriolar lymphoid sheath, (**e**) thickness of marginal zone, (**f**) thickness of thymic cortex. Values are presented as mean ± SE (*n* = 15 microscopic fields/group). (*) significant difference Vs control group, (✓) significant difference Vs HFM group at (*P* < 0.05)

Regarding the findings of histological lesion scorings, the highest score of lymphocytolysis in both the spleen and thymus was observed in HFM-exposed group. Whereas *P. peruviana* calyces extract significantly reduced the score in a dose-dependent manner compared with HFM group. The histomorphometry revealed a significant decrease in follicular diameter, marginal zone thickness, periarteriolar lymphoid sheath thickness, and thymic cortex length in HFM-exposed group compared with the control. Otherwise, treatment with PP at both doses markedly increased the entire measurements compared with HFM group. Regarding HFM + PP groups, the best improvement was noticed in the high PP-receiving group compared with those receiving the low dose (Fig. 4).

### Immunohistochemical staining

**Fig. 5:**
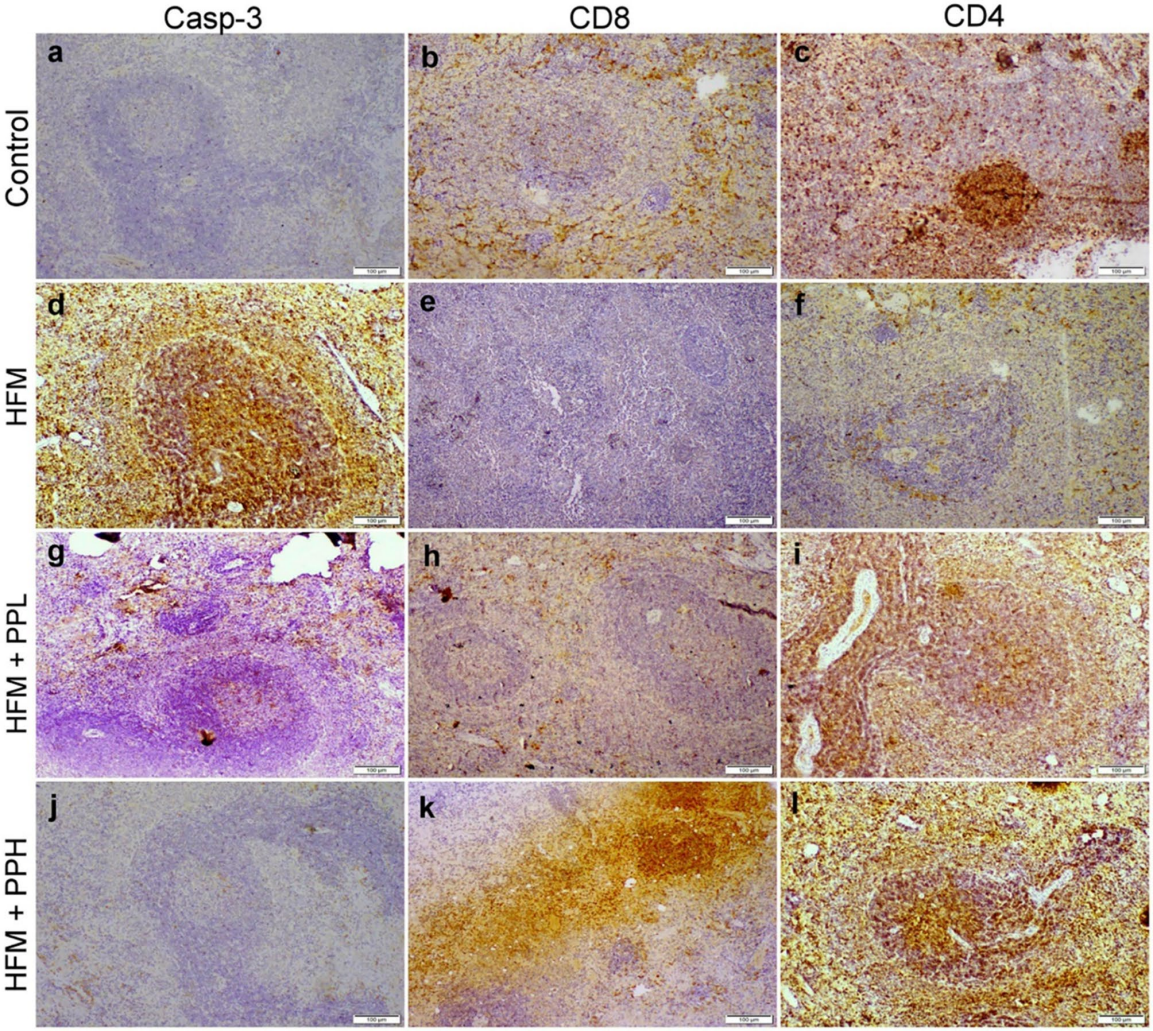
Localization of casp-3, CD4^+^, and CD8^+^ T cells in the spleen of various treatment groups. (**a-c**) control group exhibited negative casp-3 immunostaining along with huge CD8^+^ CD4^+^ T cells distributed in lymphoid follicles in white pulp and also within the red pulp. (**d-f**) HFM group displayed strong casp-3 immunostaining as well as weak CD8 and CD4 immunostaining. (**g-i**) HFM + PPL (low dose) group exhibited weak casp-3 immunostaining, moderate CD8 immunostaining, and strong CD4 immunostaining. (**j-l**) HFM + PPH (high dose) group exhibited negative c

Immunohistochemical analysis of the spleen tissues from control rats revealed no expression of caspase-3 (casp-3), while CD8 and CD4 expression was moderately positive within the normal baseline levels. Conversely, the HFM-exposed group displayed strong positive casp-3 staining, along with a significant decrease in the number of CD8 and CD4^+^ T cells. Interestingly, co-treatment with *P. peruviana* calyces extract (at either dose) alongside HFM exposure resulted in a significant decrease in casp-3 expression compared to the HFM-exposed group. While both CD8 and CD4^+^ cells markedly increased in their number compared to HFM group (Fig. 5; Table 6).

**Table 6:** Mean percentage area of casp-3, CD8, and CD4 immunopositivity in the spleen of various experimental groups

	Control	Low PP	High PP	HFM	HFM + Low PP	HFM + High PP
Casp-3%	0.5 ± 0.2	0.5 ± 0.2	0.5 ± 0.1	35.6 ± 9.6 *	15 ± 2.2 * ✓	1.8 ± 0.2 ✓
CD8%	19 ± 1.5	17.5 ± 7.5	20.4 ± 6.7	7.3 ± 5.8 *	20.1 ± 1.2 ✓	25.2 ± 0.2 * ✓
CD4%	25.5 ± 5.6	26.3 ± 6.5	25.9 ± 8.7	6.5 ± 7.2 *	27.1 ± 8.9 ✓	37.5 ± 0.2 * ✓

Values are presented as mean ± SE (*n* = 21 microscopic fields/group). (*) significant difference Vs control group, (✓) significant difference Vs HFM group at (*P* < 0.05)

## Discussion

The immune system is a complex network of events that requires meticulous regulation to function effectively without posing a threat to the body. It must possess the ability to differentiate between self and external infections and terminate an immune response once the pathogen is eradicated. Any disruption to this intricate equilibrium could have enduring implications for the organism’s survival and well-being. Certain pesticides exhibit immunomodulatory properties and may present a significant public health risk, as indicated by data from animal studies and human case reports. Consequently, our study aimed to elucidate the mechanism of HFM-induced immunotoxicity and to identify a safe approach to combat such toxicity in rats through the utilization of two distinct doses of *P. peruviana* calyces extract.

In this study, the total leukocyte count and the differential leukocyte count were employed to assess toxicity in the immune system. Bioassays revealed that HFM exerts a substantial detrimental effect on immunological cells, resulting in leucopenia, neutrophilia, and lymphocytopenia, accompanied by a notable decrease in IgM and IgG concentrations. It is postulated that the immunotoxicity of HFM stems from oxidative damage induced by free radicals generated by HFM on the primary lymphoid organs [34]. Given that the redox state and its equilibrium are pivotal in maintaining immune system homeostasis [35]. An unbalanced redox state was evaluated in this study via measuring serum TAC, and MDA concentration. HFM exposed group showed a significant increase in MDA level concurrent with a significant depletion in TAC and CAT enzymatic activity. The immune system components production was greatly limited due to oxidative stress [36]. According to reports, a key factor affecting immune cell function is the redox balance [35]. In agreement with our data, oxidative damage by HFM was previously demonstrated in the liver and kidneys as well as in the brains of rats. The rise in MDA concentration is due to the ROS-induced increase in lipid peroxidation [37]. TAC and CAT provide cellular self-defense against free radicals. The observed decrease in TAC shows that the antioxidant defense is ineffective in preventing the oxidizing effects of ROS. Increased use of both enzymes in the detoxication of superoxide radicals and H_2_O_2_ may explain the inhibition of CAT activity [38]. Accumulating oxidative stress is presumably implicated in the immunosuppressive effect and pathological mechanism of HFM–induced damage to the spleen and thymus.

From the molecular aspect, HFM could upregulate the transcription level of IL-1β, IL-6, TGF-1β, and Cox-2 genes and downregulate the transcription level of IL-10 genes in both spleen and thymus tissues. Our findings confirmed the suppressive effect of HFM on both humoral and cell-mediated immunity. Additionally, the histological changes seen in the spleen and thymus tissues are anticipated to be a result of oxidative stress. The microscopic picture of both the spleen and thymus revealed lymphoid cell depletion with extensive lymphocytolysis. Additionally, the immunostaining technique displayed strong casp-3 protein expression with a marked decrease in the number of both CD8 and CD4^+^ T cells in the spleen of the HFM group compared with the control one. We speculate that HFM can affect antigen-presenting cells (APCs), T helper (Th) cells, T cytotoxic (Tc) cells, and B cells, which can lead to immunosuppression via oxidative stress [39]. Oxidative stress causes lipid peroxidation and protein degradation, leading to lymphocytic cell necrosis and apoptosis. This may therefore exert an impact on lymphocyte growth and T/B cell-cell cooperation necessary for the generation of antibodies [40]. HFM can stimulate the release of proinflammatory cytokines such as IL-1β, and IL-6 and impair the release of anti-inflammatory cytokines IL-10. For a successful immune response, proinflammatory and anti-inflammatory cytokines must coexist in balance [41]. The oxidative stress and activation of different transcription factors may be the causes of the elevated IL-1 and IL-6 levels [42]. Anti-inflammatory cytokines are necessary to reduce this effect as a protective response since proinflammatory cytokines may intensify the inflammatory response, contributing to uncontrolled tissue damage with a significant formation of free radicals [43]. Previous research showed that IL-10 upregulation is crucial for defense against inflammation-induced damage and that its reduction indicates immunological diseases [44]. We suggest that HFM upregulates the transcription levels of TGF-1β, decreasing the number of CD4 + Th cells and inhibiting the release of IL-10. The upregulation of Cox-2 results from membrane phospholipid degradation induced by ROS, which is used as a marker for inflammation [45].

Another potential cause of immunodeficiency evoked by HFM is apoptosis. It is primarily mediated by the death receptor pathway, endoplasmic reticulum route, and mitochondrial pathway [46]. Numerous cytotoxic substances trigger programmed cell death (apoptosis) in the mitochondrial pathway by activating the caspase family of cysteine proteases. ROS increases the permeability of the mitochondrial outer membrane, causing the release of intermembrane proteins that can trigger numerous death pathways [47]. Cytochrome C (CYTC) is released into the cytoplasm when the mitochondrial membrane potential in apoptotic cells is depolarized, which enhances the permeability of mitochondrial membranes. Apoptotic bodies, APAF-1, Procaspase-9, and CYTC are coupled to activate Casp-9, which activates Casp-3 and causes lymphocytic cell death [48]. This finding is attributed to the strong casp-3 protein expression in the HFM group.

Data presented in the current study revealed a significant improvement of the redox state via inhibition of serum MDA and increase of TAC and replenishment CAT activity upon supplementation with *P. peruviana* calyces extracts in either high or low doses. In addition, administration of *P. peruviana* calyces’ extracts showed a significant immune-protective effect on HFM-exposed rats through significant elevation of TLC and lymphocytes % as well as IgG concentration. *Physalis peruviana* calyces’ extracts have the potential to be employed as a dietary antioxidant, preventing diseases induced by ROS, and ameliorating oxidative damage [49]. Other beneficial activities of *P. peruviana* calyces’ extracts are based on their antioxidant, anti-inflammatory, and antiapoptotic activities, which are most likely mediated by bioactive active compounds in the plant’s different parts [50]. The current study revealed that administration of *P. peruviana* calyces extracts with HFM improved the microscopic picture of both spleen and thymus which demonstrated normal histological appearance. The spleen of both PP-treated groups displayed weak casp-3 immunostaining and strong CD8 and CD4 immunopositivity compared with HFM group indicating the potential immunostimulant and antiapoptotic activity of *P. peruviana* calyces’ extract. Additionally, both groups receiving cotreatment with PP at either low or high dosage levels demonstrated a decrease in the mRNA levels of IL-1β, IL-6, Cox-2, and TGF-1β genes, along with an increase in IL-10 levels compared with HFM group. These findings indicated the anti-inflammatory and immunostimulant effect of *P. peruviana* calyces extracts on HFM-exposed rats.

UPLC-MS analysis of *P. peruviana* calyces’ extract revealed a diverse metabolite profile, with sucrose esters being the most abundant class. Acyl sucrose sugars are regarded as the main protective secondary metabolites constituting the resinous matter of the inner parts of many *Physalis* species calyces [51]. This class of compounds exhibits numerous interesting biological properties such as anti-inflammatory, multidrug resistance modulatory, anti-skin aging, antidiabetic, antibacterial, and immunomodulatory activities [12, 13, 52]. Notably, these compounds have demonstrated significant anti-inflammatory activity and may hold promise in regulating the immune system given the link between inflammation and immune dysfunction. By mitigating inflammation, sucrose esters could potentially enhance the body’s ability to combat pathogens [10, 12, 53, 54]. This prevalence suggests they may be a critical component of the calyces’ observed bioactivity. The product ion chromatogram of *P. peruviana* calyx extract revealed the abundance of a large group of metabolites previously described as functionalized sucrose esters with isobutanoyl, methylbutanoyl, pentenoyl, octanoyl, nonanoyl, decanoyl, and dodecanoyl substituents [55], which are mostly abundant within the retention time range from 0.61 to 14.13 min. The UPLC-MS/MS chromatogram allowed for the tentative identification of 15 acyl sucrose sugars, with a fragmentation pattern consisting of the [M-H]^−^ ion and the initial loss of one R group, such as isobutanoyl (C_4_H_6_O, 70 Da), pentenoyl (C_5_H_6_O, 82 Da), 2-metylbutanoyl (C_5_H_8_O, 84 Da), octanoyl (C_8_H_14_O, 126 Da), nonanoyl (C_9_H_16_O, 140 Da), decanoyl (C_10_H_18_O, 154 Da) and dodecanoyl (C_12_H_22_O, 182 Da) and ascribed as mono-, di-, tri- and tetrasubstituted sucroses. Although hydroxyl groups at C6, C1’, and C6’ positions of the sucrose moiety appeared to be more reactive due to lower steric hindrance, the C2, C3, C1’, and C3’ hydroxyl groups were described in the literature and reported to be the most favored positions for esterification by saturated fatty acids in *P. peruviana* [51]. Therefore, the identified ester residues and the positions of all substituents in the disaccharide structure were tentatively assigned according to MS/MS data along with data reported in the literature. For instance, peak (S1) [M-H]^−^at *m/z* 411.16 (C_16_H_27_O_12_^−^) yielded base peak fragment ion at *m*/*z* 341 [M-H-70] ^–^ due to isobutanoyl group (70 Da) loss was tentatively identified as monosubstituted *O*-isobutanoylsucrose (Supplementary Fig. S1) [55]. Among the identified disubstituted acyl sucrose sugar, di-*O*-isobutanoylsucrose with deprotonated molecular ion at *m/z* at 481.11 (C_20_H_33_O_13_^−^) was annotated in peak (S2) based on *m/z* at 463 due to water molecule loss (18 Da), *m/z* 393 due to isobutanoyl group loss (70 Da), and base peak ion at *m/z* 323 [Sucrose-H] due to additional isobutanoyl group loss (Supplementary Fig. S2) [55]. *O*-Decanoyl-*O*-isobutanoylsucrose with deprotonated molecular ion at *m/z* at 565.29 (C_26_H_45_O_13_^−^) was detected in peak (S4) which yielded fragment ions at *m/z* 547, 477, and 323 corresponding to water (18 Da), isobutanoyl (70 Da), and decanoyl (154 Da) loss, respectively, and *O*-isobutanoyl-*O*-octenoylsucrose identified in peak (S3) with [M-H]^−^ at *m*/*z* 535.26 (C_24_H_39_O_13_^−^) provided fragment ion at *m/z* 517 due to water loss (18 Da) and base peak ion at *m/z* 393 [M-H-H_2_O-acyl fatty acid] ^−^ corresponding to octenoyl moiety loss (124 Da) (Supplementary Figs. S3 & S4) [55]. Concerning trisubstituted acyl sucrose, peak (S6) [M-H]^−^ at *m*/*z* 647.33 (C_31_H_51_O_14_^−^) showed its main fragments at *m*/*z* 493 [M-H-154] ^−^ from the loss of decanoyl group (154 Da) and *m*/*z* 411 [M-H-82-154]^−^ due to loss of methylbutenoyl group (82 Da), and *m*/*z* 341 due to loss of isobutanoyl (70 Da), thus it was identified as *O*-decanoyl-*O*-isobutanoyl-O-(2 methylbutenoyl)sucrose [44]. Peak (S8) with [M-H]^−^ at *m*/*z* 695.35 yielded the [M-H]^−^ ion at 649 (C_31_H_53_O_14_^−^) due to the loss of the formic acid (46 Da) and main fragment ions at *m*/*z* 495, 411, and 341 due to loss of decanoyl (154 Da), methylbutanoyl (84 Da), and isobutanoyl (70 Da) groups, respectively, was ascribed as *O*-decanoyl-*O*-isobutanoyl-*O*-(2-methylbutanoyl)sucrose (Supplementary Fig. S5) [44]. Peak (S14) with [M-H]^−^ at *m*/*z* 733.37 (C_37_H_65_O_14_^−^) yielded main fragment ions at *m*/*z* 645 [M-H-18-70]^−^ due to the loss of one water molecule followed by isobutanoyl group loss (70 Da), *m/z* 463 [M-H-18-70-decanoyl]^−^, and 323 [M-H-18-70-decanoyl-nonanoyl]^−^ due to the loss of dodecanoyl (182 Da) and nonanoyl (140 Da) respectively, was identified as *O*-dodecanoyl-*O*-isobutanoyl-*O*-nonanoylsucrose [55]. *O*-Decanoyl-di-*O*-isobutanoylsucrose was detected in peak (S7) with [M-H]^−^ at *m*/*z* 635.24 (C_30_H_51_O_14_^−^) gave main fragment ion at *m/z* 481 due to decanoyl loss (152 Da) and was further fragmented to yield *m/z* 411 corresponding to isobutanoyl loss (70 Da), and 342 [sucrose-decanoyl-2 isobutanoyl-H]^−^ [55]. Di-*O*-isobutanoyl-*O*-octanoylsucrose with [M-H]^−^ at *m*/*z* 607.13 (C_28_H_47_O_14_^−^) was assigned in peak (S5), which exhibited characteristic fragment ions at *m/z* 481 post the loss of Octanoyl moiety (126 Da), and *m/z* at 393 due to loss water molecule (18 Da) and isobutanoyl group (70 Da) [55]. Meanwhile, tetrasubstituted acyl sucrose sugars were detected in peaks (S9, S10, S12 & S13). Peak (S12) with [M-H]^−^ ions at *m/z* 691.4 (C_33_H_55_O_15_^−^) constituted three isobutanoyl groups and acyl fatty acid, where it showed fragment ions detected at *m/z* 621 and 551 due to consecutive loss of two isobutanoyl moieties, *m/z* 411. due to loss nonanoyl moiety (140 Da), and main fragment ion at *m/z* 341due to additional isobutanoyl loss (70 Da), hence, it was identified as *O*-nonanoyl- tri-*O*-isobutanoylsucrose isomers [55]. Similarly, *O*-octanoyl-tri-*O*-isobutanoyl-sucrose adduct was found in peak (S13) with [M-H]^−^ at *m*/*z* 732.34 yielded the [M-H]^−^ ion at 677 (C_32_H_53_O_15_^−^) due to the loss of the formic acid (46 Da) and showed characteristic fragment ions at *m/z* 607 [M-H-70]^−^, 537 [M-H-70-70]^−^, 411 [M-H-70-70-126]^−^ corresponding to consequential loss of two isobutanoyl moiety (70 Da) and octanoyl moiety (126 Da), and base peak fragment ion at *m/z* 342 [56]. *O*-Decanoyl-*O*-diisobutanoyl-*O*-(2-methylbutenoyl) sucrose was identified in peak (S10) with [M-H] ^−^ at *m/z* 717.31781 (C_35_H_57_O_15_^−^) and characteristic fragment ions at *m/z* 563, 481, 411, 342 corresponding to decanoyl (154 Da), methylbutenoyl (82 Da) and two isobutanoyl moiety (70 Da), respectively (Supplementary Fig. S6). Supporting this activity is the existing research on other *Physalis* species. A previous study on *P. angulata* revealed that sucrose esters are the key contributors to anti-inflammatory effects, leading to the potential immunomodulatory role of these compounds in *P. peruviana* as well [13]. While the correlation between sucrose esters and immunomodulation is promising, further research is necessary to elucidate the specific mechanisms by which sucrose esters within *P. peruviana* calyces could modulate the immune system. Isolation and testing these specific sucrose esters in vitro and in vivo models could provide valuable insights into their immunomodulatory properties.

Moreover, the major identified withanolides are recognized to display a wide variety of emerging biological activities including antimicrobial, antitumor, anti-inflammatory, cytotoxic, hepatoprotective, antidiabetic, as well as immunomodulatory activities [57–59]. The negative ionization mode allowed for the tentative identification of eight isobaric withanolide-type compounds classified according to their different hydroxy and epoxy positions and complementary information provided by MS/MS product ion spectra. The fragmentation pattern of withanolides is mainly characterized by multiple water molecule losses (− 18 Da) and the cleavage of C-17-substituted lactone moiety (Lac) to afford the [M-H-Lac]^−^ product ion as the prevalent pathway. Further dehydration fragments were detected in MS/MS spectra due to several losses of water molecules from the ergostane moiety [M − H−Lac]^−^ [59]. This pattern is well-reported to be attributed to the occurrence of 4/5-hydroxyl and 5,6/6,7-epoxide groups in the ergostane moiety, whose removal might be favored due to extended conjugation generated with the C1-α-*β*-unsaturated keto group [56], and aided in further information regarding the hydroxylation of the lactone moiety, as this part is removed as a neutral entity, while the ergostane moiety remains charged. For example, a neutral loss of 140 Da corresponds to a hydroxylated lactone, while a loss of 124 Da corresponds to non-hydroxylated lactones [55]. The previously reported withanolide D and E derivatives were detected in peak (W7) and peak (W6) with [M-H]^−^ at *m*/*z* 469.2607 (C_28_H_37_O_6_^−^) and [M-H]^−^ at *m*/*z* 485.2550 (C_28_H_37_O_7_^−^) with prevalent fragments ions at *m*/*z* 345 and *m*/*z* 361 [M-H-124]^−^, respectively, corresponding to the loss of the lactone residue (124 Da) (Supplementary Figs. W1 & W2) [44]. For instance, peak (W2) [M-H]^−^ at *m/z* 519.26 (C_28_H_39_O_9_^−^) yielded fragment ion at *m/z* 379 [M − H−Lac]^−^ due to neutral loss of hydroxylated lactone moiety (140 Da) along with other lower intensity fragments ions 361 [379-H_2_O]^−^, 343 [361-H_2_O]^−^ corresponding to the loss of water was tentatively identified as 2,3-dihydro-27-hydroxy-4*β*-hydroxywithanolide E isomer (Supplementary Fig. W3). Similarly, peak (W4) with [M-H] − at *m*/*z* at 519.2601 (C_28_H_39_O_9_^−^) was annotated as 24,25-dihydro-4,27-dihydroxylatedwithanolide E isomer with the characteristic loss of hydrogenated lactone moiety (142 Da) affording fragment ion at *m*/*z* 377, which was further dehydrated to yield daughter ions at *m/z* 359 [377-H_2_O]^−^, and 341 [359-H_2_O-H_2_O]^−^ (Supplementary Fig. W4) [55]. Meanwhile, the previously identified 17, 27-dihydroxylatedwithanolide D isomer was assigned in peak (W3) [M-H]^−^ 501.2494 (Supplementary Fig. W5) [55], which showed an initial water loss at *m/z* 483 followed by characteristic loss of lactone moiety (124 Da) in a fragment at *m*/*z* 359 [M-H-H_2_O Lac-]^−^ corresponding to non-hydroxylated lactone moiety, while the previously identified 2,3-dihydro-27-hydroxylated withanolide D was found in peaks (W5) with [M-H]^−^ at *m*/*z* 487.2701 (C_28_H_39_O_7_^−^), which showed water molecule loss at *m/z* 469 and intense fragment ion at m/z 345 due to neutral loss of lactone moiety (124 Da) (Supplementary Fig. W6).

*P. peruviana* is also found to be rich in hydroxycinnamates (HCDs). HCDs are a class of polyphenolic compounds that are well-documented for their antioxidant properties and are frequently encountered in edible plants [60]. Research suggests that HCDs may offer various health benefits, including antimicrobial, anticancer, and antigenotoxic properties, likely due to their potent antioxidant capacity [61]. Hydroxycinnamates (HCDs) were among the major secondary metabolites tentatively identified in *P. peruviana*, represented by 10 cinnamoyl derivatives. Peak (H1) with [M-H]^−^ at *m/z* 163.04, and fragment ion *m/*z 119 corresponding to [M-H-COO]^−^ was identified as *p*-coumaric acid [62] (Supplementary Fig. H1), while peak (H6) with [M-H]^−^ at *m/z* 325.09158 displayed a neutral loss of hexose moiety (162 amu) yielding *p*-coumaric acid fragment ion at *m/z* 163, hence, it was assigned as *p*-coumaric acid-*O*-hexoside [63]. Peak (H7) with [M-H]^−^ at *m/z* 337.09235 exhibited a similar MS^2^ fragmentation pattern to 5-acyl-hydroxy cinnamoyl quinic acid with main fragment ion at 191 *m/z* and secondary ion at *m/z* 173 was annotated as 5-*p*-coumaroylquinic acid [64]. Caffeic acid was detected at peak (H4) at *m/z* 179.03468 [M-H]^−^ with dominant fragment ion at *m/z* 135 due to decarboxylation (44 Da loss) [65] (Supplementary Fig. H2). Two caffeoylquinic acid isomers were detected in peaks (H2 and H3) with [M-H]^−^ at *m/z* 353.08707. For instance, peak (H2) showed base peak fragment ion at *m/z* 191 corresponding to quinic acid, and secondary ions at *m/z* 179 and 135 corresponding to caffeic acid and decarboxylated caffeic acid, respectively, was ascribed as 3-*O*-caffeoylquinic acid. While peak (H3) provided the MS^2^ base peak ion at *m/z* 173, which originated from quinic acid dehydration, suggesting substitution at the C-4 position of caffeoyl moiety, consequently, it was identified as 4-*O*-caffeoylquinic acid [64]. Peak (H5) was annotated as 3-*O*-feruoylquinic acid with a deprotonated molecular ion at *m/z* 367.10281 and base peak fragment ions at *m/z* 193 corresponding to deprotonated ferulic acid and 134 [ferulic acid-H-CO_2_-CH_3_] [66]. Likewise, peak (H9) showed a deprotonated molecular ion at *m/z* 355.10324 with consequent neutral loss of hexose (162 amu) to yield base peak fragment ion at *m/z* 193 corresponding to ferulic acid was assigned as ferulic acid-*O*-hexoside. Sinapoyl-*O*-hexoside was identified in peak (H8) with [M-H]^−^ at *m/z* 385.11398 with subsequent neutral loss of hexose moiety (162 amu) to produce base peak fragment at *m/z* 223 corresponding to sinapic acid, which was further fragmented to yield secondary ions at *m/z* 205 and 179 resulting from water loss from a hydroxyl group and carbon dioxide from a carboxyl group [67] (Supplementary Fig. H3). Peak (H10) gave a deprotonated molecular ion at *m*/*z* 471.1414 [M − H]^−^ with fragment ions at *m*/*z* 323 [M-cinnamic acid − H]^−^, *m*/*z*309 [M − fructosyl − H]^−^, *m*/*z* 179 [glucose − H]^−^ or [fructose-H]^−^, *m*/*z* 161[glucose − H_2_O]^−^ or [fructose − H_2_O−H]^−^ which are following the fragmentation pattern of sibirioside A [68].

Organic acids are primary metabolites that greatly influence the organoleptic and sensory properties of many edibles in flavor, color, and aroma [69]. Considering the better ionization of these carboxyl-containing compounds, UPLC-MS utilizing negative mode facilitates the detection of six different organic acids. Malic acid was detected in peak (O1) with deprotonated molecular ion at *m/z* 133.01434 and base peak fragment ion at *m/z* 115 corresponding to [M − H−H_2_O] ^−^ (Supplementary Fig. O1), while citric acid was identified in peak (O3) with deprotonated molecular ion at *m/z* 191.01981 and characteristic fragment ion at *m/z* 111 corresponding to [M − H−CO_2_ − 2H_2_O]^−^ [70]. Peak (O2) was annotated as succinic acid with a deprotonated molecular ion at m/z 117.01958 and product ions at m/z 99 [M − H−H_2_O] − and 73 [M − H−CO_2_]− [71] (Supplementary Fig. O3). Protocatechuic acid was identified in peak (O4), which showed a deprotonated molecular ion at *m/z* 153.01920 and base peak fragment ion at *m/z* 109 [M-H-CO_2_]^−^, while benzoic acid was detected at peak (O5) with [M-H]^−^at *m/z* 121.0296 and main fragment ion at *m/z* 77 [M-H-CO_2_]^−^ [72].

Flavonoids and Lignans are polyphenolic secondary metabolites widely distributed in many edible plants and detected in *P. peruviana* calyces’ extract. They are well-reported to exert numerous health benefits including antibacterial, antiviral, antioxidant, immunomodulatory, anti-inflammatory, and anticarcinogenic properties, as well as other biological activities [8, 73]. UPLC-MS in negative ionization mode led to the detection of one flavan-3-ol and four flavanol glycosides. A (epi)catechin was identified in peak (F1) with [M-H]^−^ 289.06973 and fragment ions at *m/z* 271 [M-H-H_2_O]^−^, 253 [(273-H_2_O)] ^−^, 243 [(273-CO)] ^−^, 229 [(273-C_2_H_2_O)]^−^, 225 [(273-H_2_O-CO)]^−^. The other identified flavonoid glycosides were *O*-glycosides exhibiting 162, 146, and 308 amu neutral losses, corresponding to the cleavage of hexose, deoxyhexose, and combined loss of these sugars, respectively, and releasing the characteristic aglycon ion [74]. Peak (F5) with a [M-H]^−^at *m/z* 593.15179 is characterized by neutral loss of rutinosyl moiety (306 KDa) and base peak product ion at *m/z* 285 corresponding to kaempferol aglycon; moreover, the co-existence of production at *m/z* 284 [kaempferol-H]^−^ strongly suggested the linkage of glycoside at 3-OH position, hence it was ascribed as kaempferol-3-*O*-rutinoside [75] (Supplementary Fig. F1). Kaempferol-3-*O*-hexoside was assigned to peak (F6) at m/z 447.09329 and yielding fragment ions at *m/z* 285, and 284 resulted from a neutral loss of hexose moiety, 255 [kaempferol-CH_2_O] ^−^, and 227 [kaempferol- CH_2_O-CO] ^−^ [76] (Supplementary Fig. F2). Quercetin-3-*O*-hexoside was identified in peak (F4) with a deprotonated molecular ion at *m/z* 463.08859 and diagnostic quercetin aglycon fragment ion at *m/z* 301 and 300 due to neutral hexosyl moiety loss by 162 amu, while quercetin-3-*O*-rutinoside (rutin) was found in peak (F3) with [M-H]^−^at *m/z* 609.15 and fragment ions at *m/z* 463 [M-H-rhamnose] ^−^ which was further fragmented to yield quercetin aglycon base peak secondary ions at *m/z* 301 [M-H-rhamnosyl-glucosyl] ^−^ together with fragment ion at *m/z* 300, suggesting 3-*O*-glycosidic linkage [77]. Similarly, quercetin-*O*-hexosyl-rhamnosyl hexoside was detected in peak (F2) at *m/z* 771.19690 and produced fragment ions at *m/z* 609 [M-H-hexosyl], 463 [M-H-hexosyl-rhamnosyl], and 301 [M-H-hexosyl-rhamnosyl-hexosyl]^−^ [78].

Lignans have received considerable attention due to their proven pharmacological benefits, including anticancer, anti-inflammatory, antiviral, and antioxidant activities [79]. The mass spectral data of *P. peruviana* calyces in negative ionization mode allowed the tentative identification of three furano lignans. Lariciresinol was detected in peak (L2) with [M-H]^−^at *m/z* 359.15036 and characteristic fragment ion at *m/z* 329 due to the loss of one hydroxymethyl group as formaldehyde [M-H-30]^−^ [80]. At the same time, lariciresinol-*O*-hexoside was identified in peak (L1) at *m/z* 521.20306 showing base peak fragment ion of lariciresinol aglycon at *m/z* 359 resulting from neutral hexose unit loss and a secondary ion at *m/z* 329 [81] (Supplementary Fig. L1). Additionally, peak (L3) at *m/z* 557.24561 yielded the main fragment ion at *m/z* 395 [M-H-hexose]^−^ and secondary ions at *m/z* 377 and 359 due to consecutive loss of two water molecules was tentatively identified as dihydroxy lariciresinol-hexoside.

Compared to hydrophilic primary metabolites exemplified by acyl sugars, phenolics, and flavonoids. The obtained MS^n^ spectra revealed the presence of lipophilic hydroxylated fatty acid derivatives. It is worth noting that hydroxylated fatty acids have gained appreciable attention due to their promising antifungal, antibacterial, cytotoxic, and anti-inflammatory activities [82, 83]. Late in the chromatographic run, several hydroxylated fatty acids were tentatively identified based on their characteristic losses of 18, 36, and 44 amu corresponding to the cleavage of H_2_O, 2H_2_O, and COOH [84]. Monohydroxylated fatty acid as hydroxyhexadecandioic acid was assigned to peak (A1) with a deprotonated molecular ion at *m/z* 301.2019 (Supplementary Fig. A1) [85]. While dihydroxy-octadecenoic acid and trihydroxy-octadecenoic acid were identified in peaks (A4) and (A3) with [M-H]^−^ at *m/z* 313.23819 and 329.23346 corresponding to molecular formulas (C_18_H_33_O_4_^−^) and (C_18_H_33_O_5_^−^), respectively (Supplementary Fig. A2 & A3) [86]. Moreover, another trihydroxylated fatty acid was detected in peak (A2) with deprotonated molecular ion at m/z 325.2020 (C_18_H_29_O_5_) ^−^ and showed a four amu difference from that of peak (A3), indicating the presence of two extra double bonds, hence it was identified as trihydroxy-octadecatrienoic acid (Supplementary Fig. A4).

This study paves the way for developing new strategies to combat the harmful effects of pesticides on human health. Further research is needed to isolate the active compounds in the extract, conduct more in vivo studies, and explore their potential as a natural immune system support. Additionally, future studies should focus on elucidating the mechanisms of action at a clinical level and identifying specific compounds that could be used in nutraceuticals for immune disorders.

## Conclusion

This study presents novel findings about the action mechanism of HFM-induced immunotoxicity and revealed the possible protective role of *P. peruviana* calyces’ extract (PP) against such toxicity. HFM negatively affected both humoral and cell-mediated immunity in rats via decreasing total and differential lymphocytic counts, immunoglobulin levels, and altering the histological structure of both spleen and thymus tissues. Oxidative stress plays a major role in HFM-mediated immunotoxicity via initiating inflammation and apoptosis in the immune organs and altering their structure and functions. *P. peruviana* calyces extract exhibited immunostimulant, anti-inflammatory, and antiapoptotic activities. LC-MS/MS profiling of *P. peruviana* calyces ethanolic extract tentatively allowed the identification of various metabolite classes, with acyl sucrose esters, withanolides, and flavonoids regarded as the major metabolites, more likely to contribute to the observed biological effects.

## Electronic supplementary material

Below is the link to the electronic supplementary material.

Supplementary Material 1

## Data Availability

All data generated or analysed during this study are included in this published article [and its supplementary information files].

## Abbreviations

APAF-1: Apoptotic bodies
APCs: Antigen-presenting cells
Casp-3: Caspase-3
CAT: Catalase
CD-4: Cluster of differentiation 4
CD-8: Cluster of differentiation 8
Cox-2: Cyclooxygenase-2
CYTC: Cytochrome C
DPPH: 2,2-diphenyl-1-picrylhydrazyl
HCDs: Hydroxycinnamates
HeLa: Human cervical cancer
HFM: Hexaflumuron
IL-6: Interleukin-6
IL-8: Interleukin-8
INOS: Inducible nitric oxide synthase
L929: Murine fibroblast cells
MCP-1: Monocyte chemoattractant protein-1
MDA: Malondialdehyde
MUC-2: Oligomeric mucus/gel-forming
NF-kB: Nuclear factor kappa B
PGE2: Prostaglandin E2
PP: Physalis peruviana L. calyces extract
RAW 264.7 cells: The murine macrophage cell line
ROS: Reactive oxygen species
TAC: Total antioxidant capacity
TBARS: Thiobarbituric acid reactive substance
Tc cells: T cytotoxic
TFF-3: Trefoil factor 3
TGF-1b: Transforming growth factor beta 1
Th cells: T helper cells
TNF-α: Tumor Necrosis Factor-alpha
UPLC-MS/MS: Ultra-high-performance liquid chromatography
