# Supplementary material for: Physalis peruviana calyces extract ameliorate oxidative stress, inflammation, and immune loss in rats-exposed to hexaflumuron
Source: BMC Complement Med Ther. 2025 Jan 22;25:21. doi: 10.1186/s12906-025-04750-z (PMC11756176; doi:10.1186/s12906-025-04750-z)
Supplement: Supplementary file 1 — Supplementary Material 1 [file 12906_2025_4750_MOESM1_ESM.pdf]

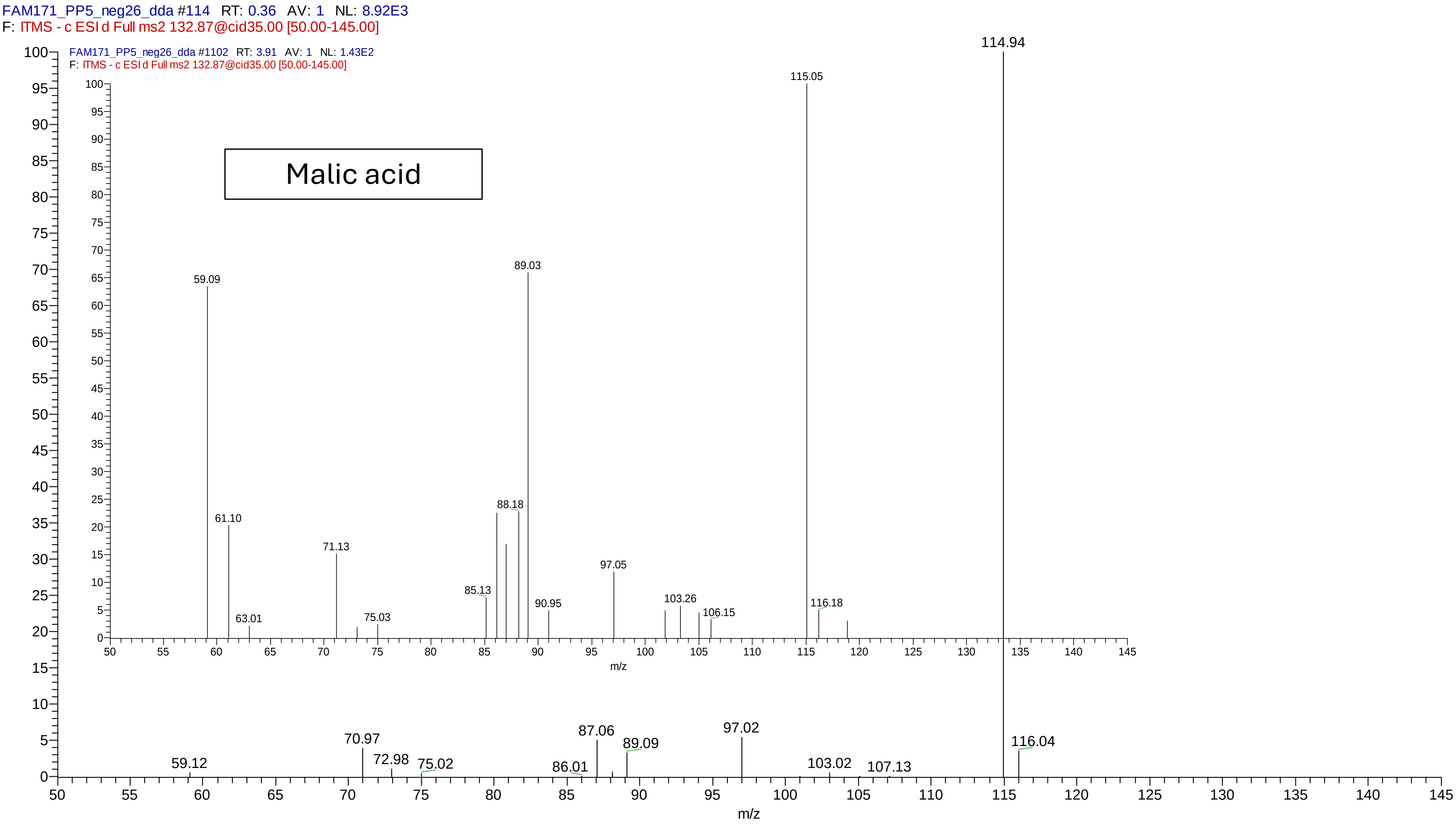

Fig.O1: MS/MS fragmentation spectrum of peak O1

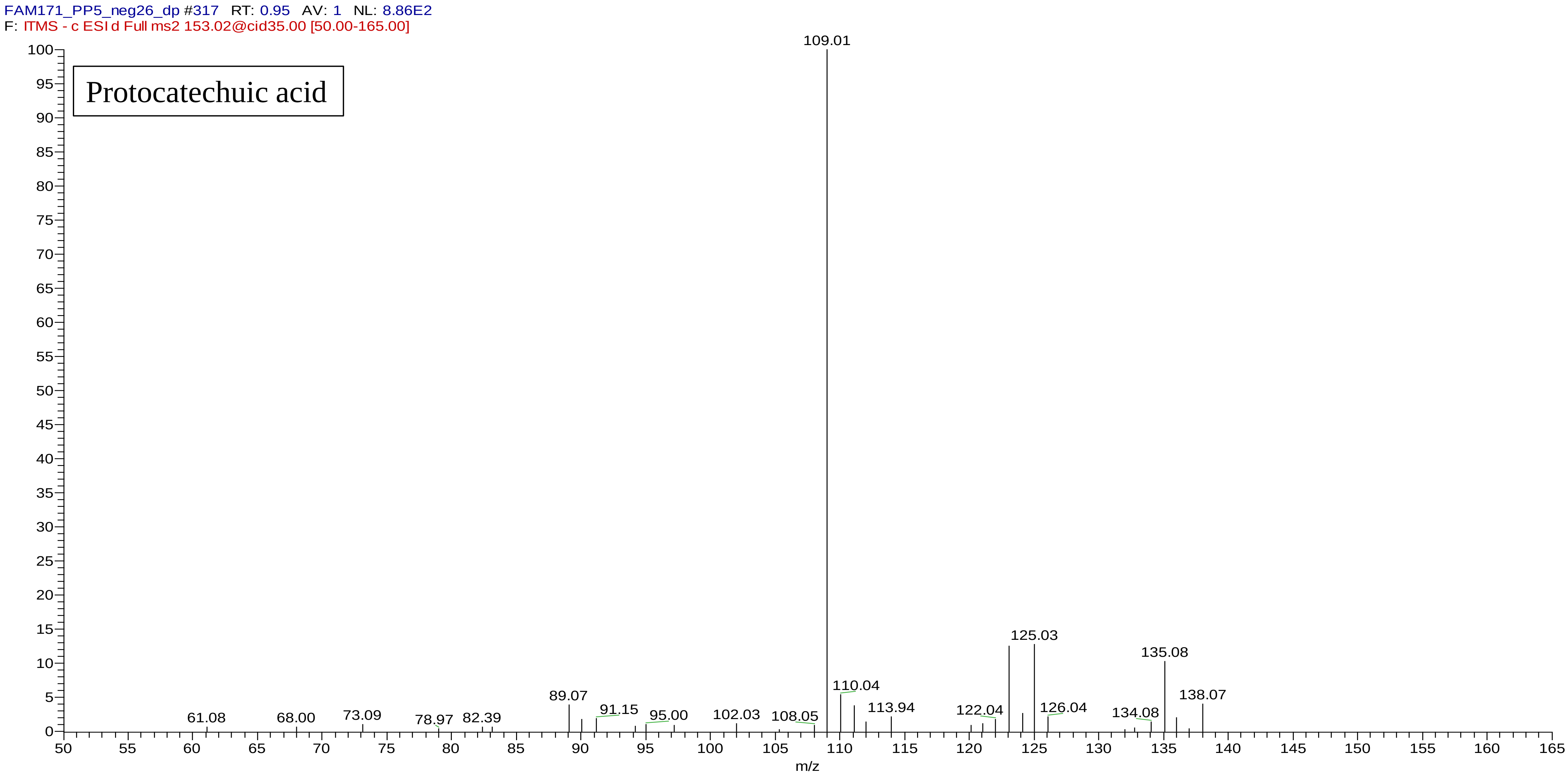

Fig.O2: MS/MS fragmentation spectrum of peak O4

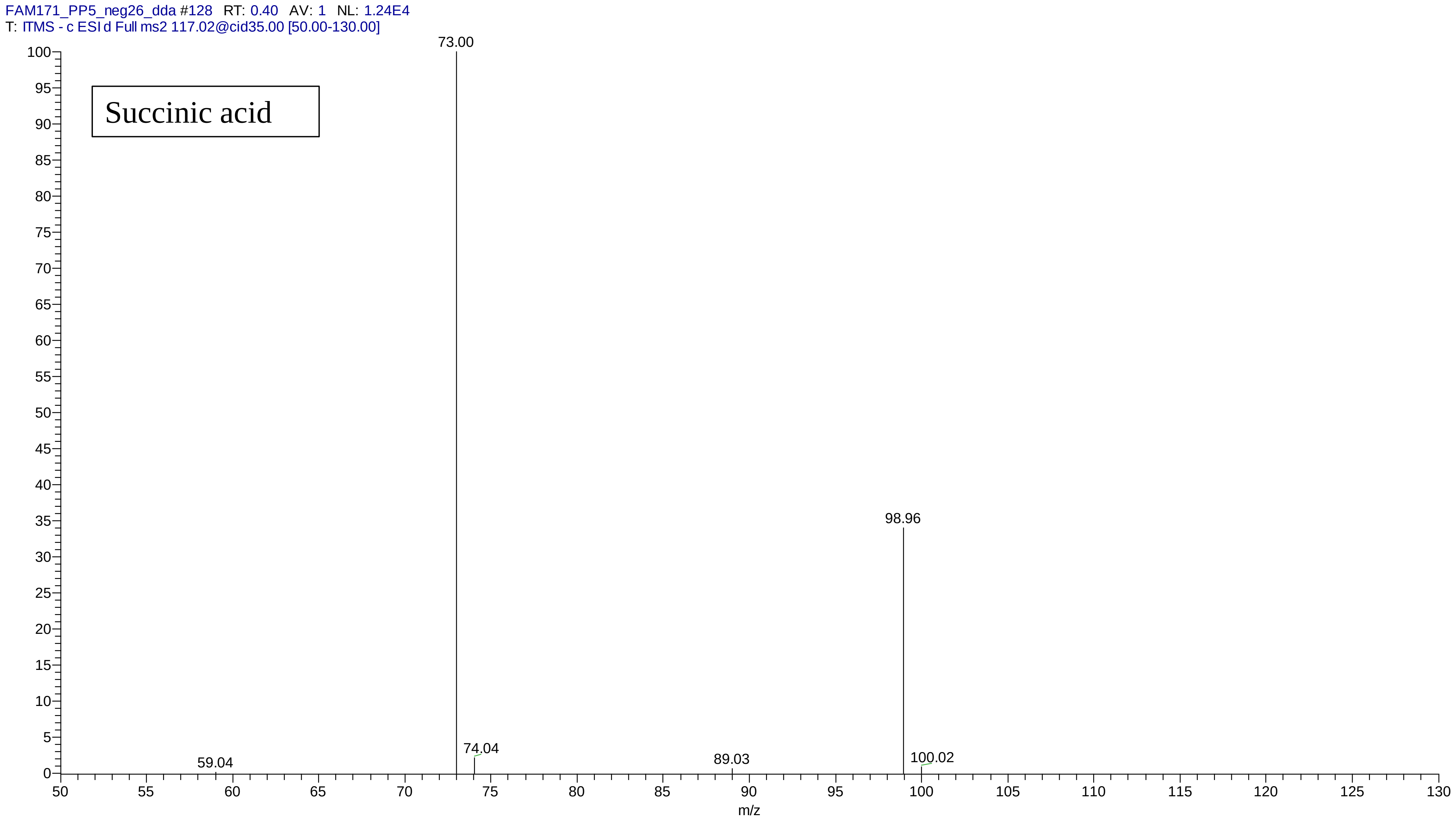

Fig.O3: MS/MS fragmentation spectrum of peak O2

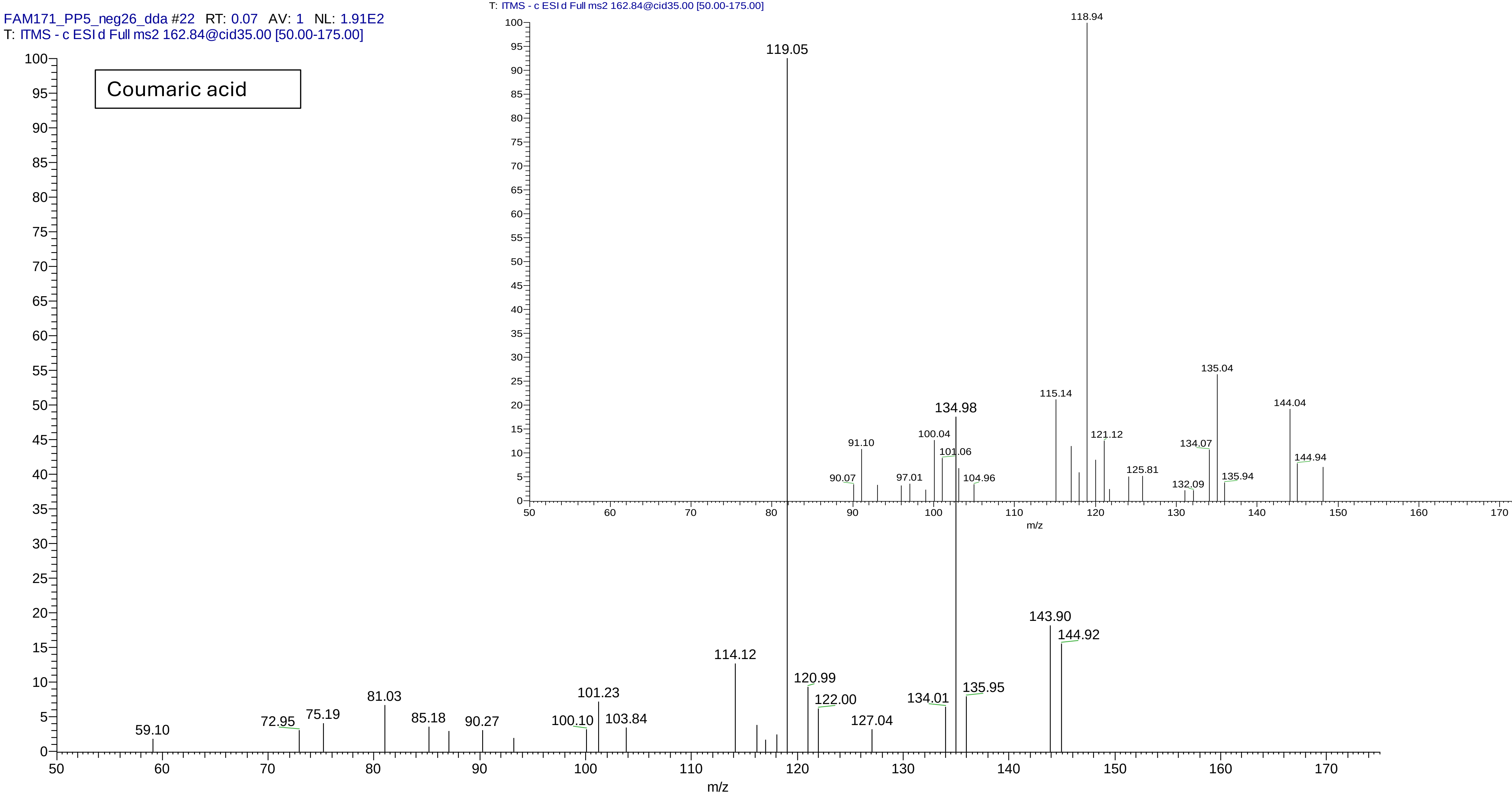

Fig.H1: MS/MS fragmentation spectrum of peak H1

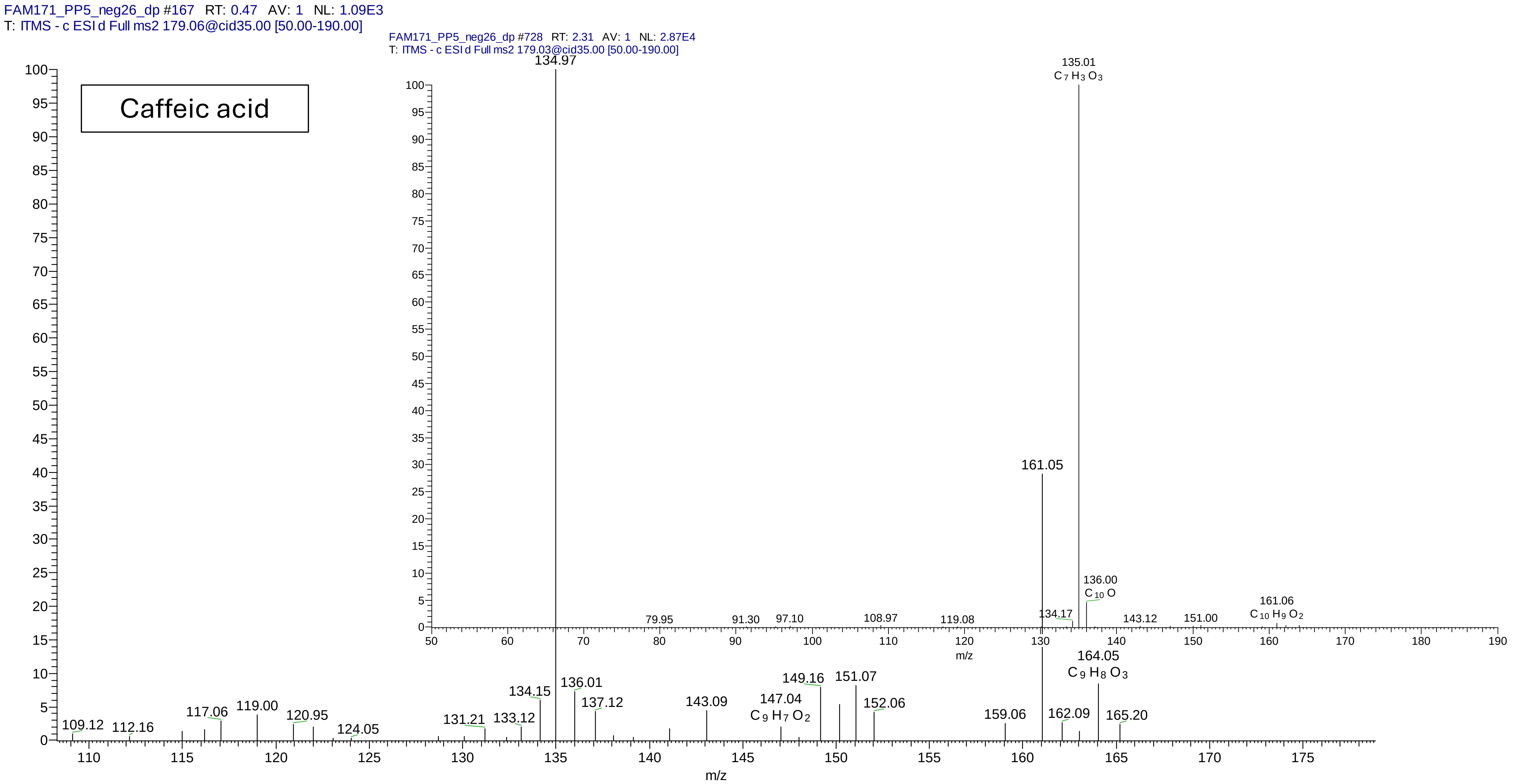

Fig.H2: MS/MS fragmentation spectrum of peak H4

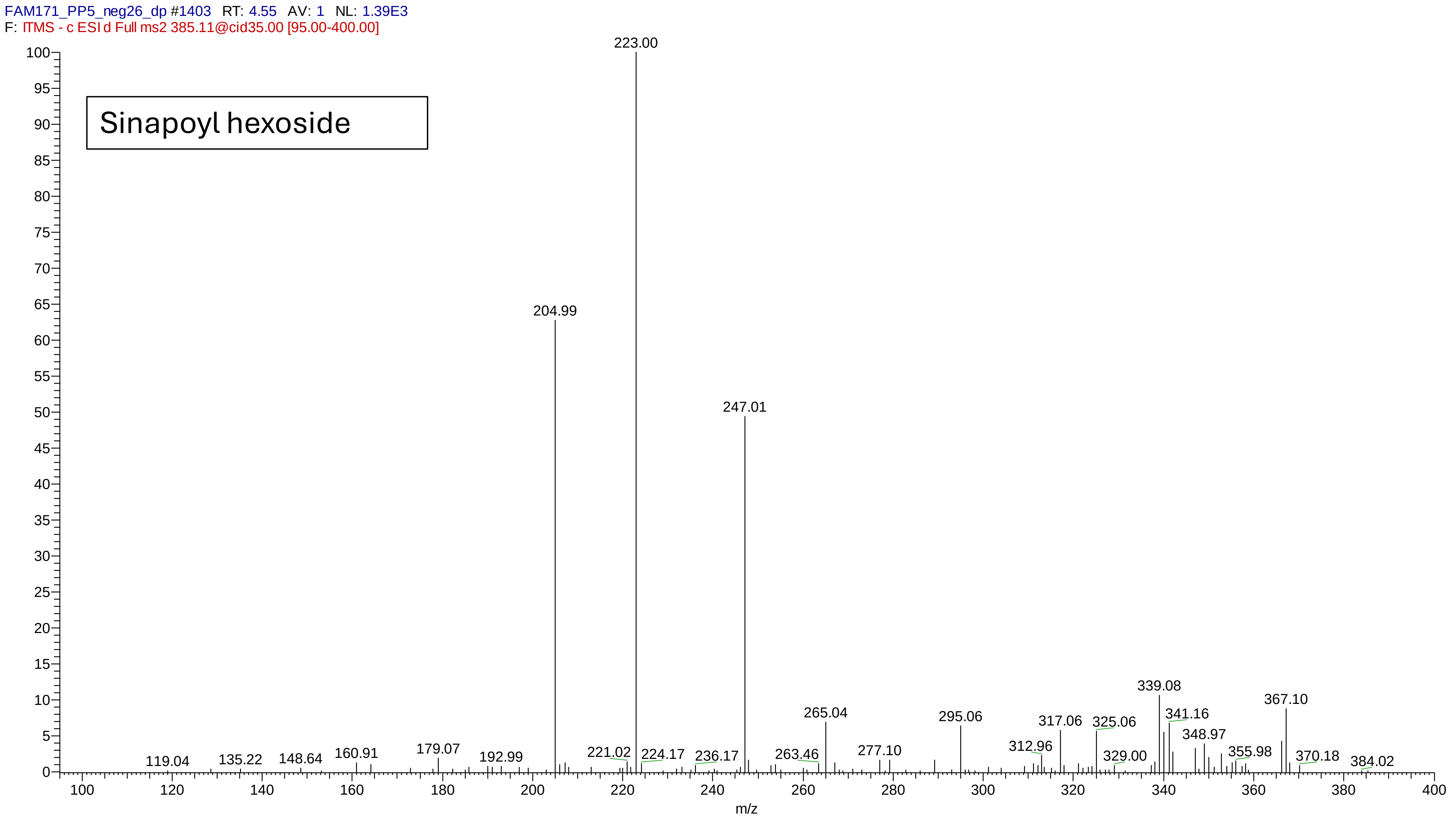

Fig.H3: MS/MS fragmentation spectrum of peak H8

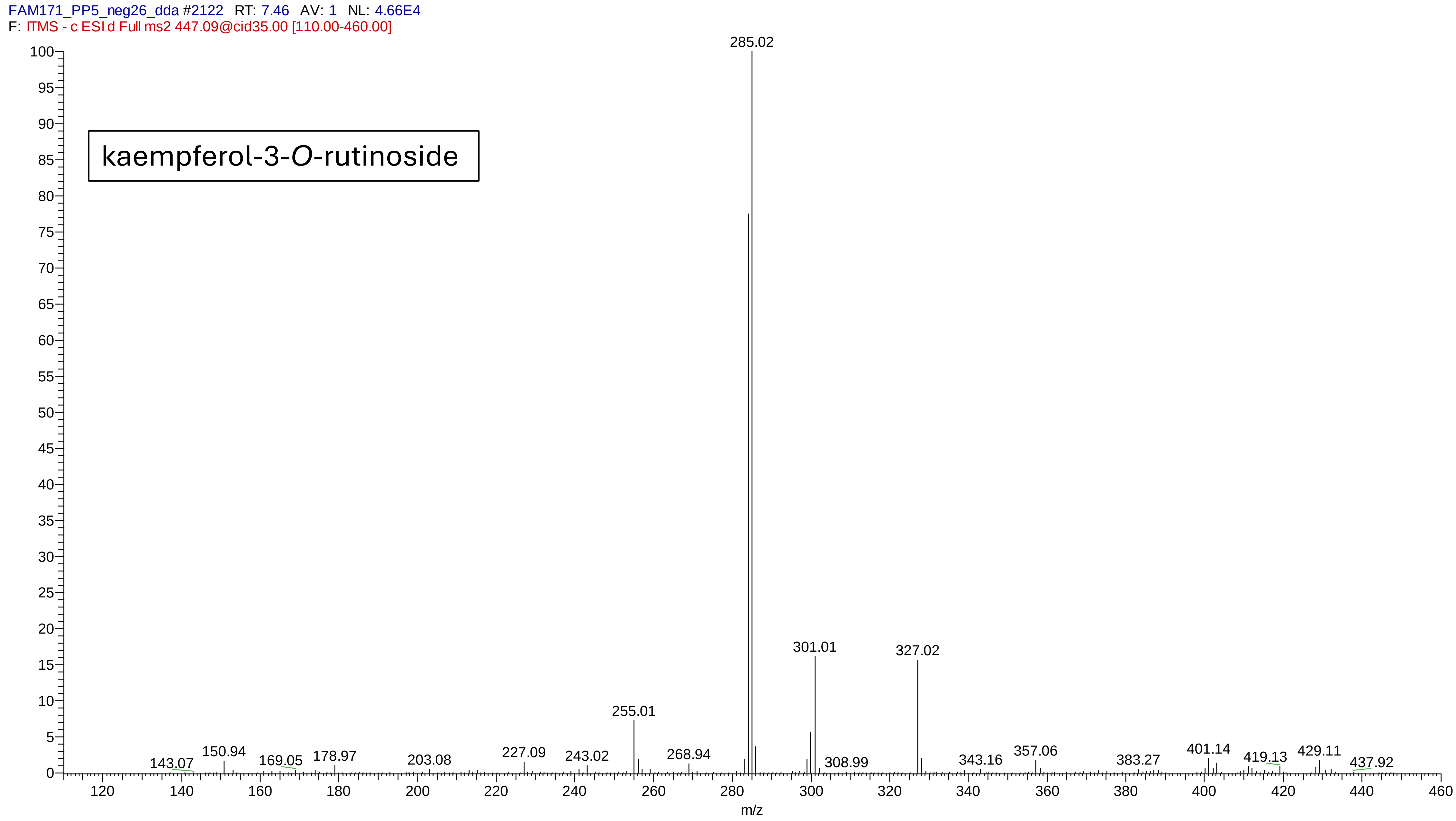

Fig. F1: MS/MS fragmentation spectrum of peak F5

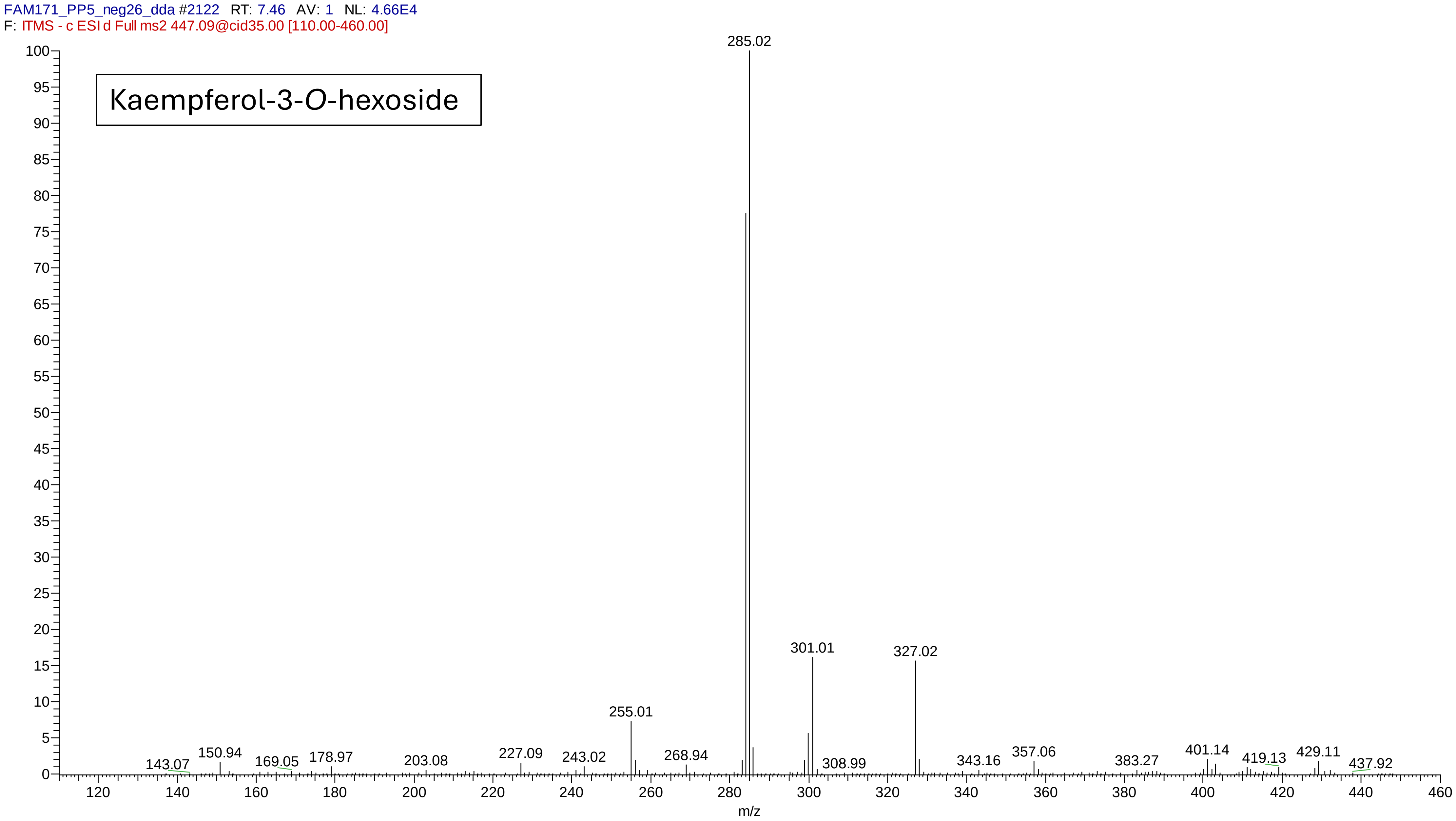

Fig. F2: MS/MS fragmentation spectrum of peak F6

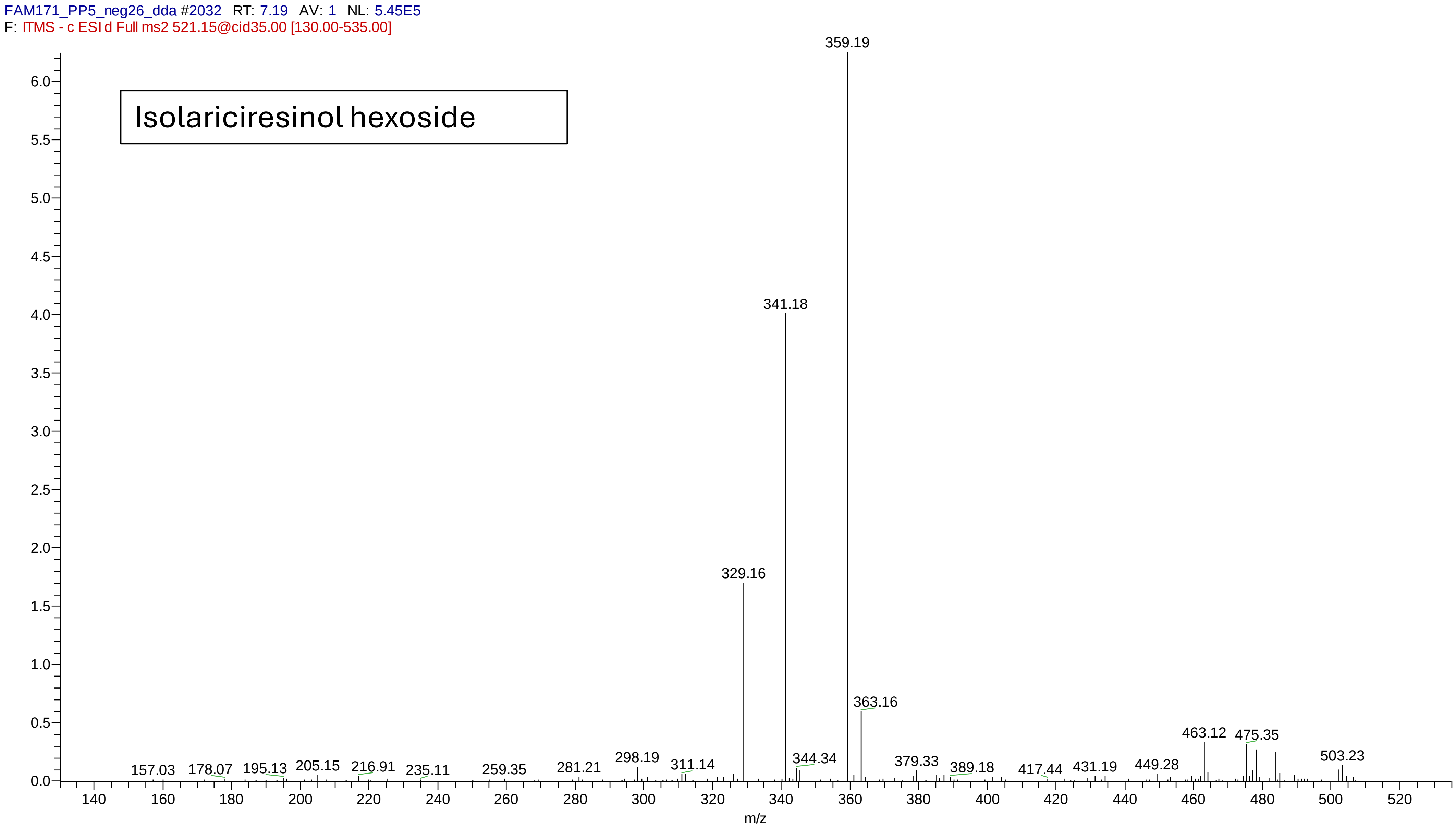

Fig. L1: MS/MS fragmentation spectrum of peak L1

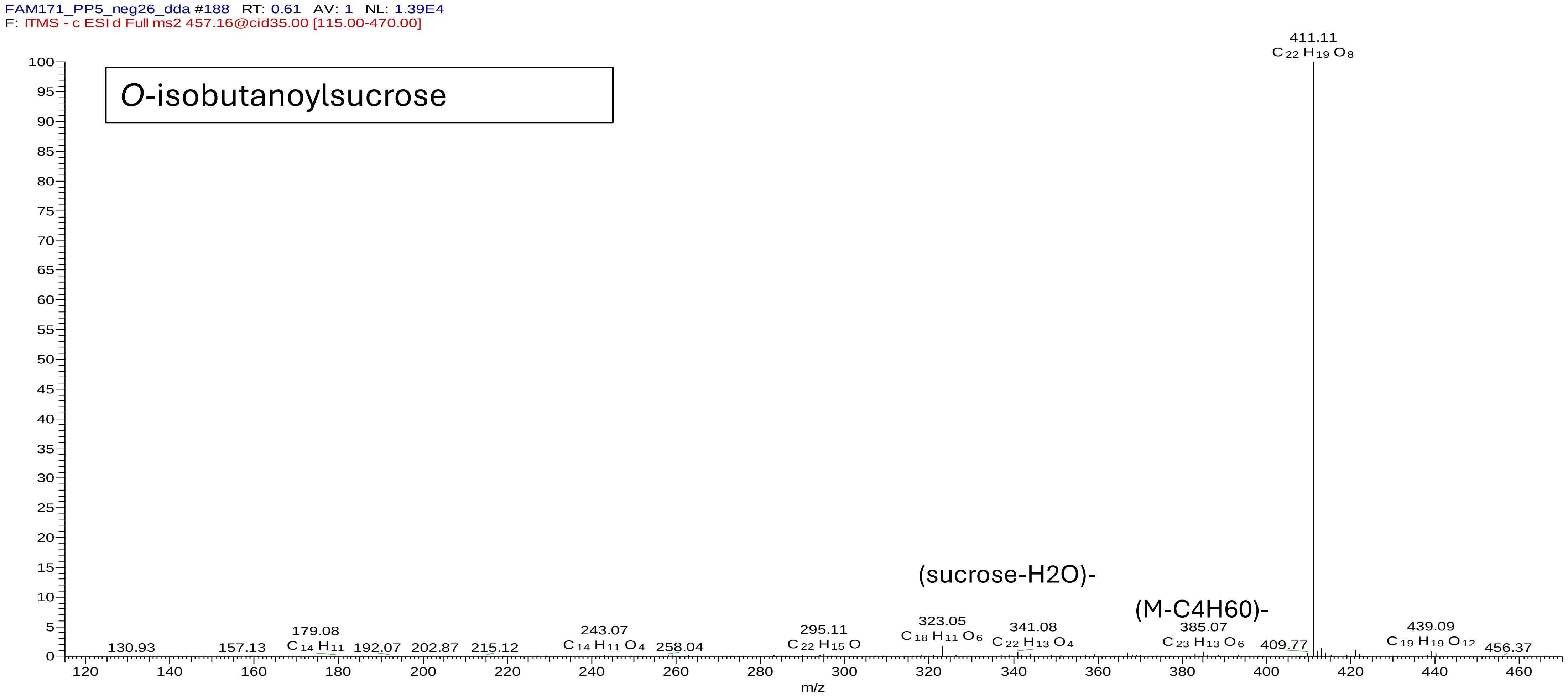

Fig.S1: MS/MS fragmentation spectrum of peak S1

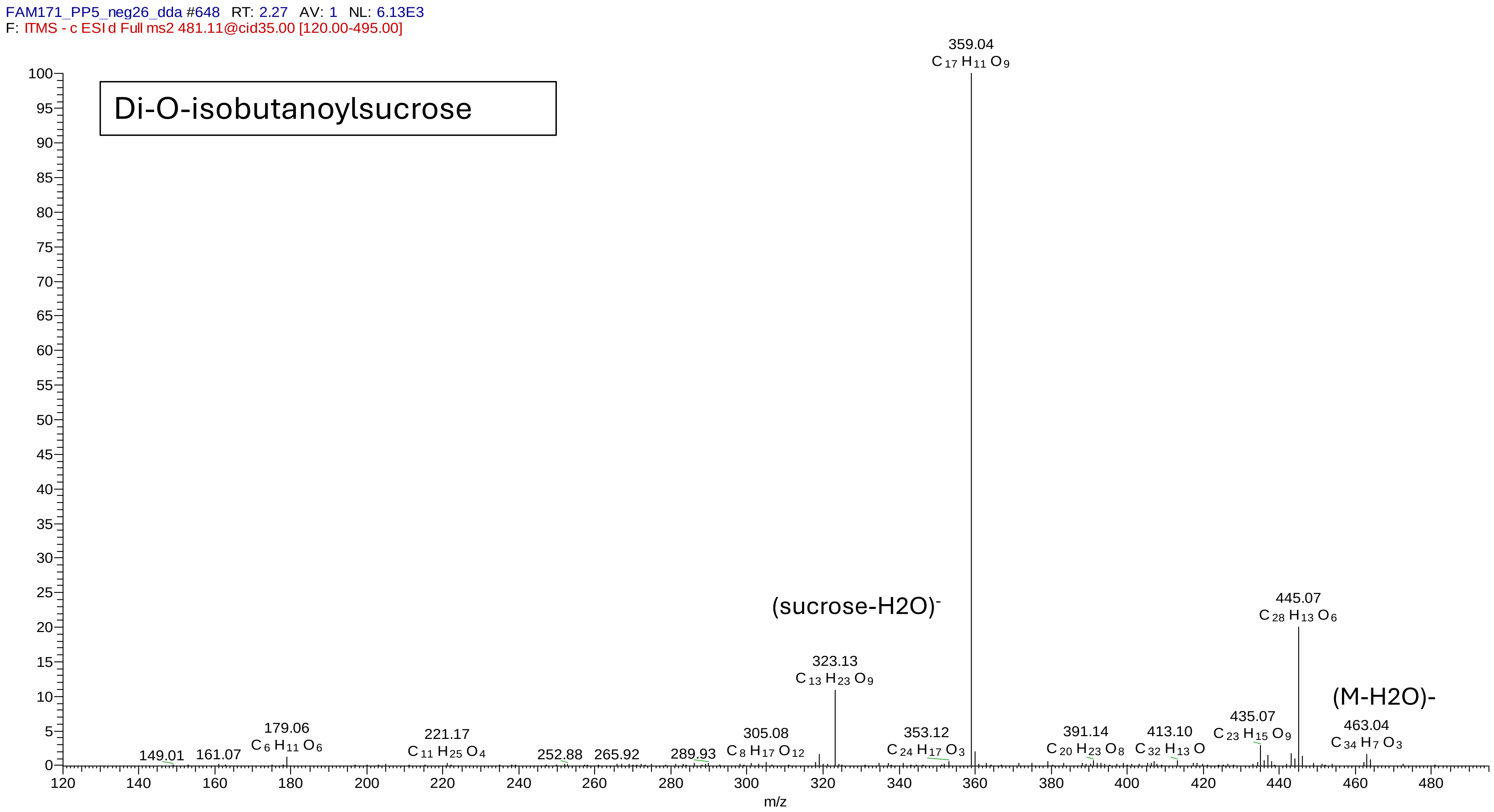

Fig.S2: MS/MS fragmentation spectrum of peak S2

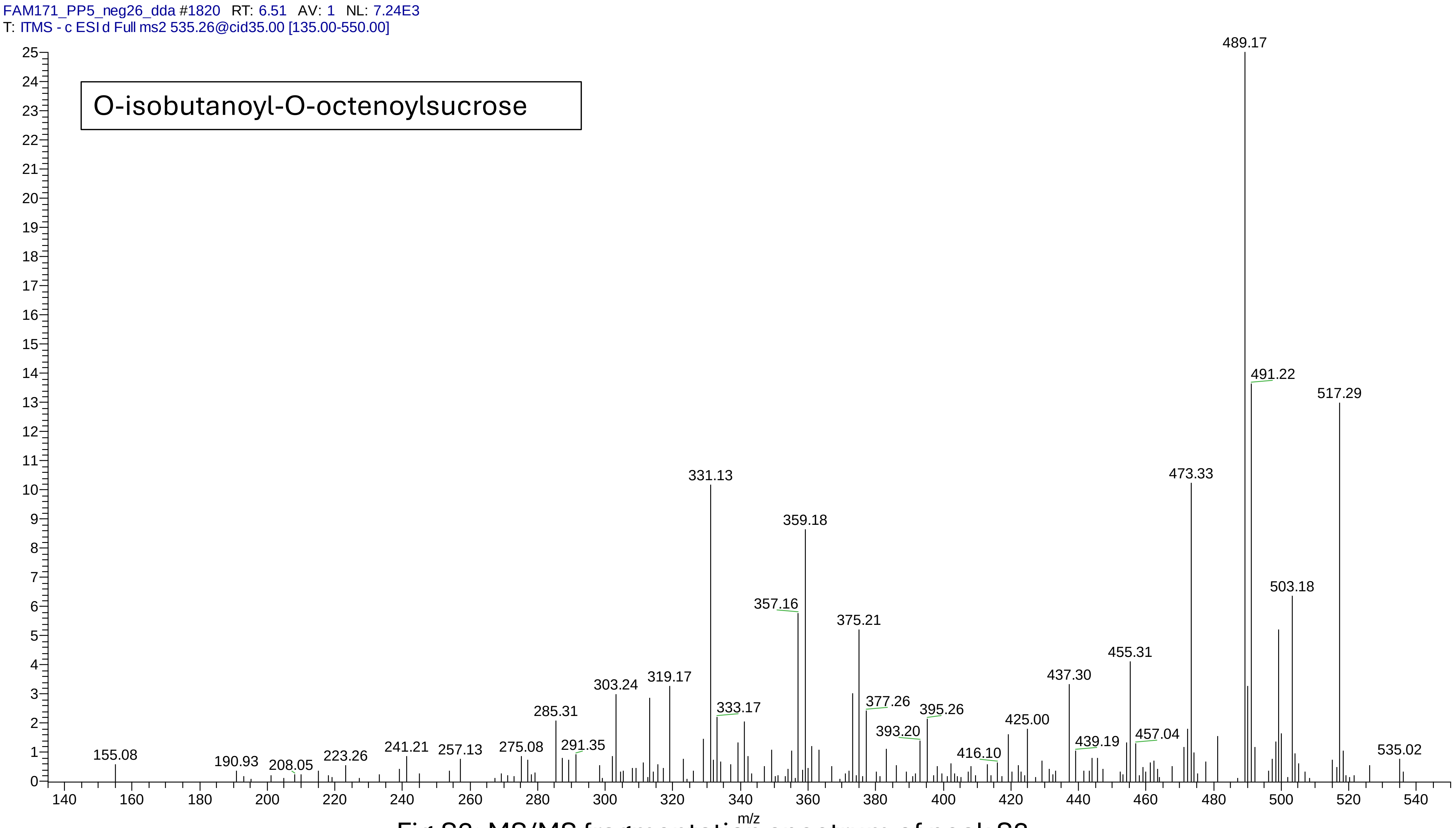

Fig.S3: MS/MS fragmentation spectrum of peak S3

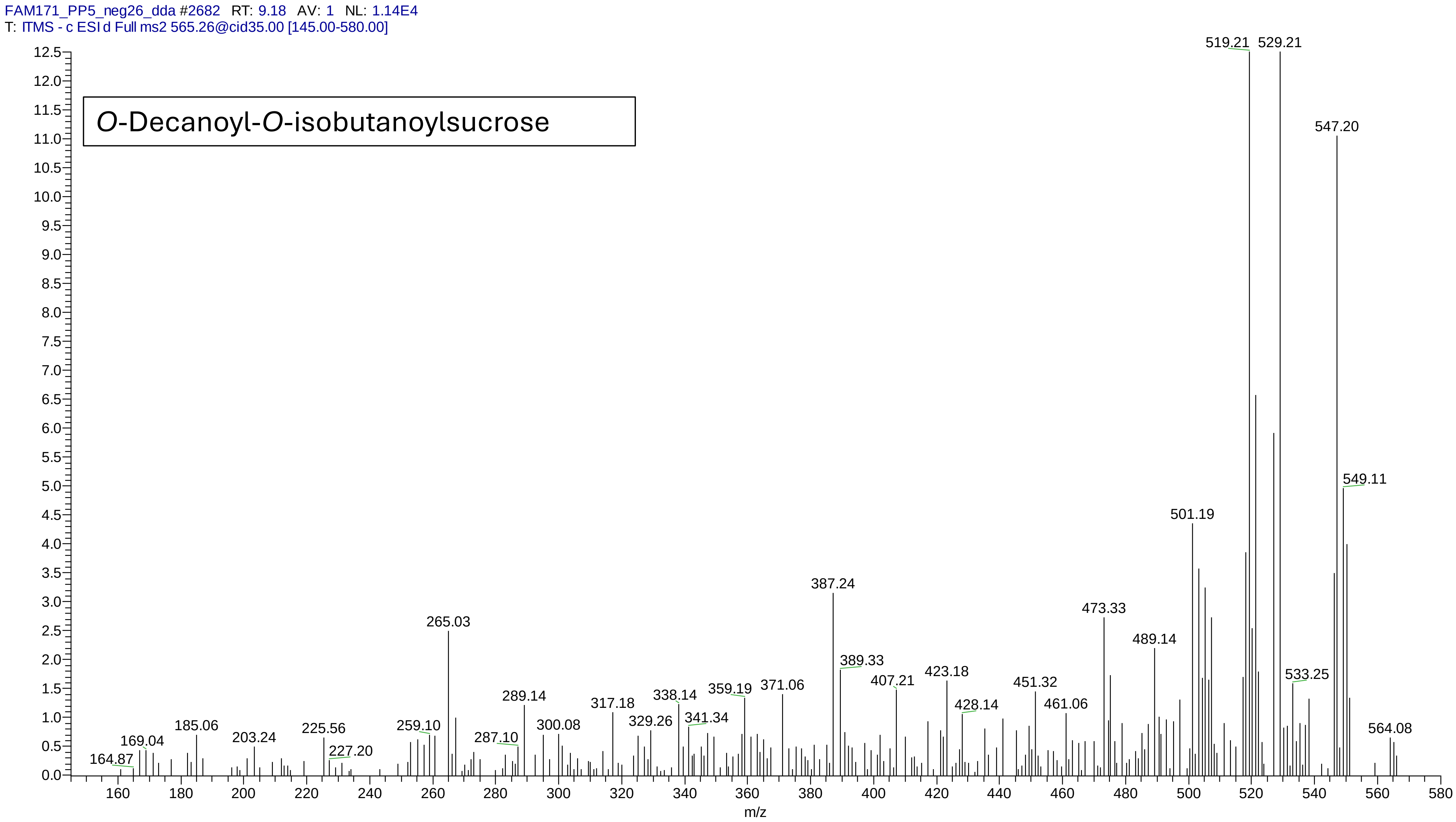

Fig.S4: MS/MS fragmentation spectrum of peak S4

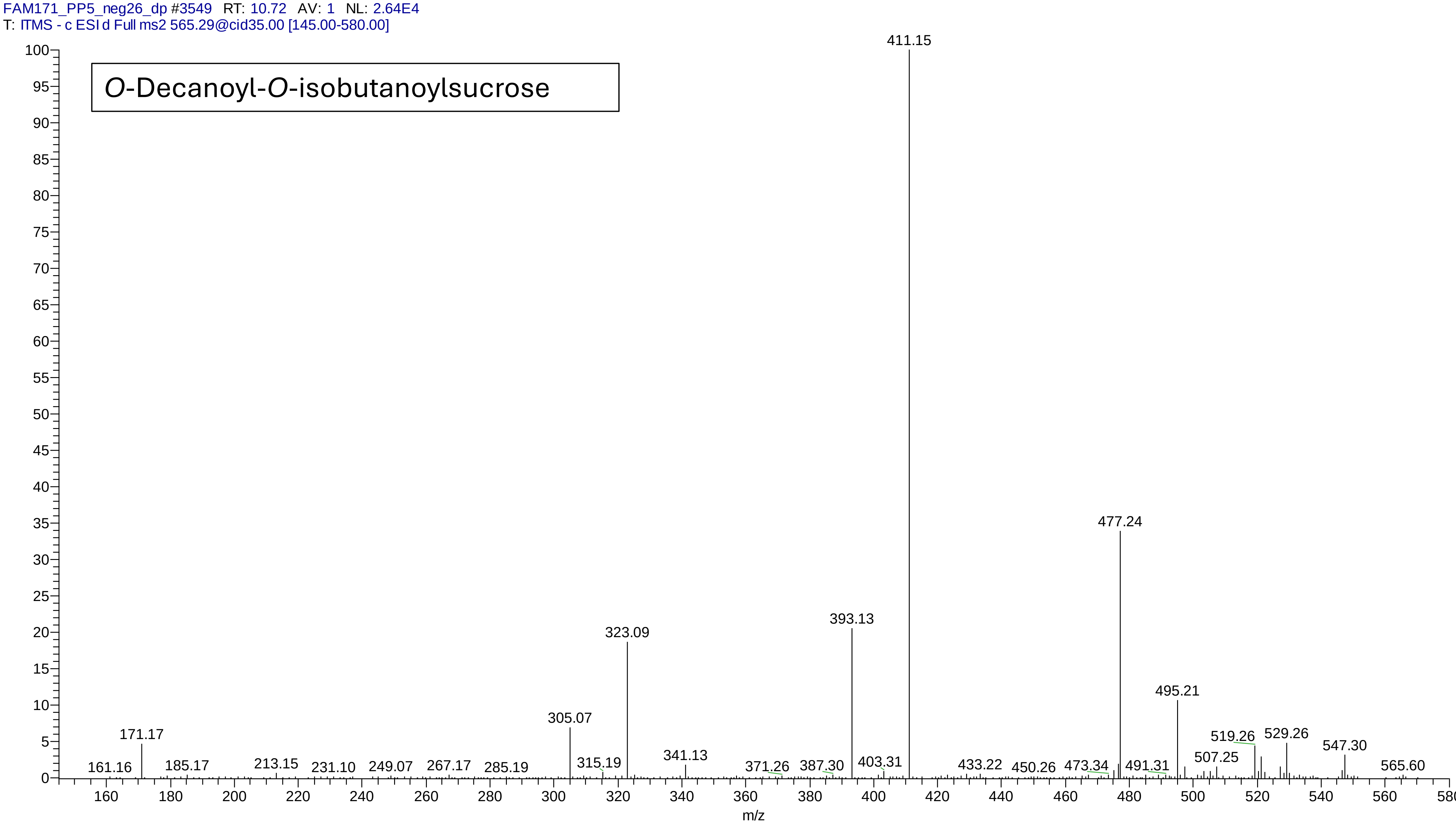

Fig.S4: MS/MS fragmentation spectrum of peak S4

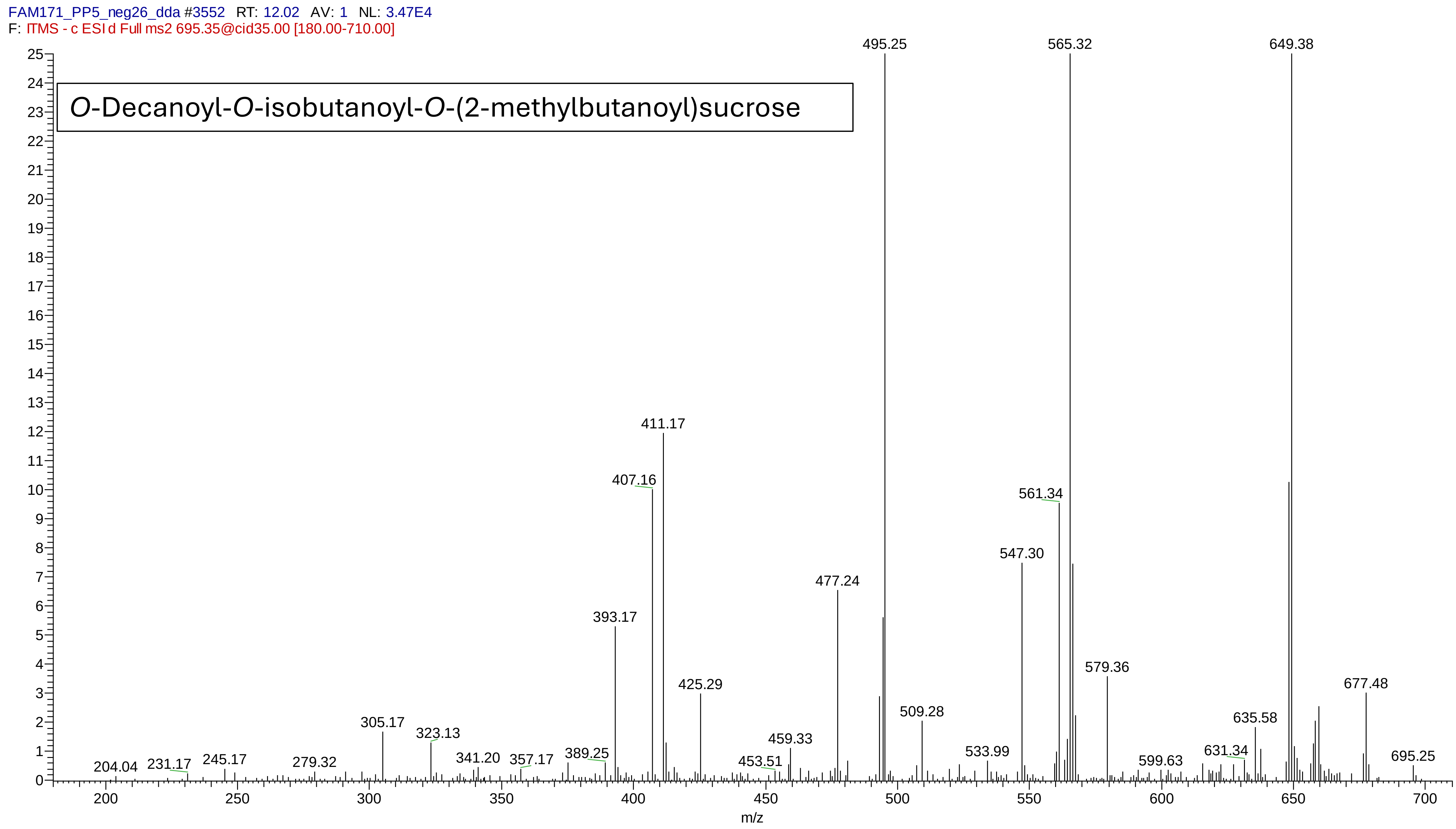

Fig.S5: MS/MS fragmentation spectrum of peak S8

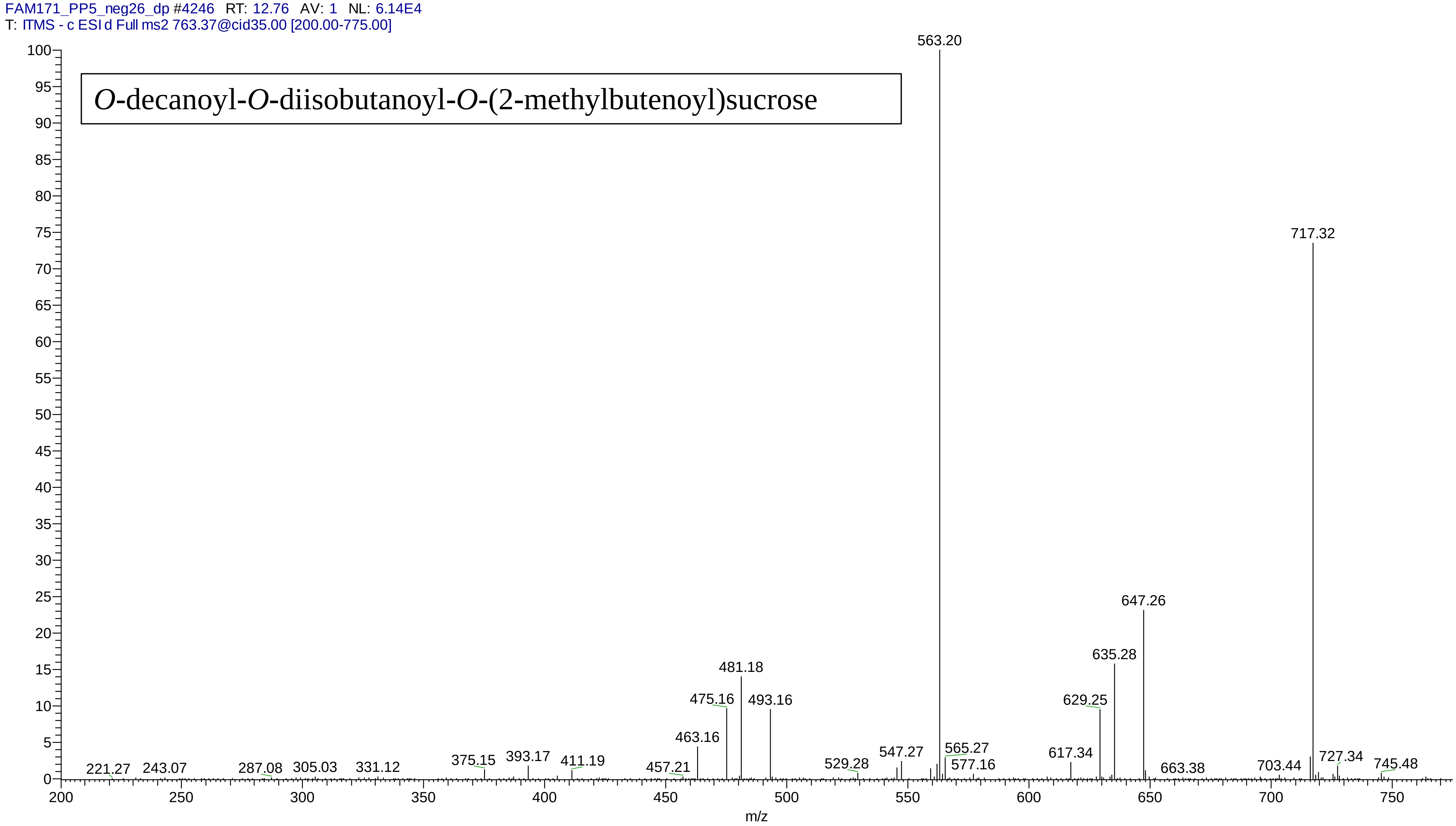

Fig.S6: MS/MS fragmentation spectrum of peak S10

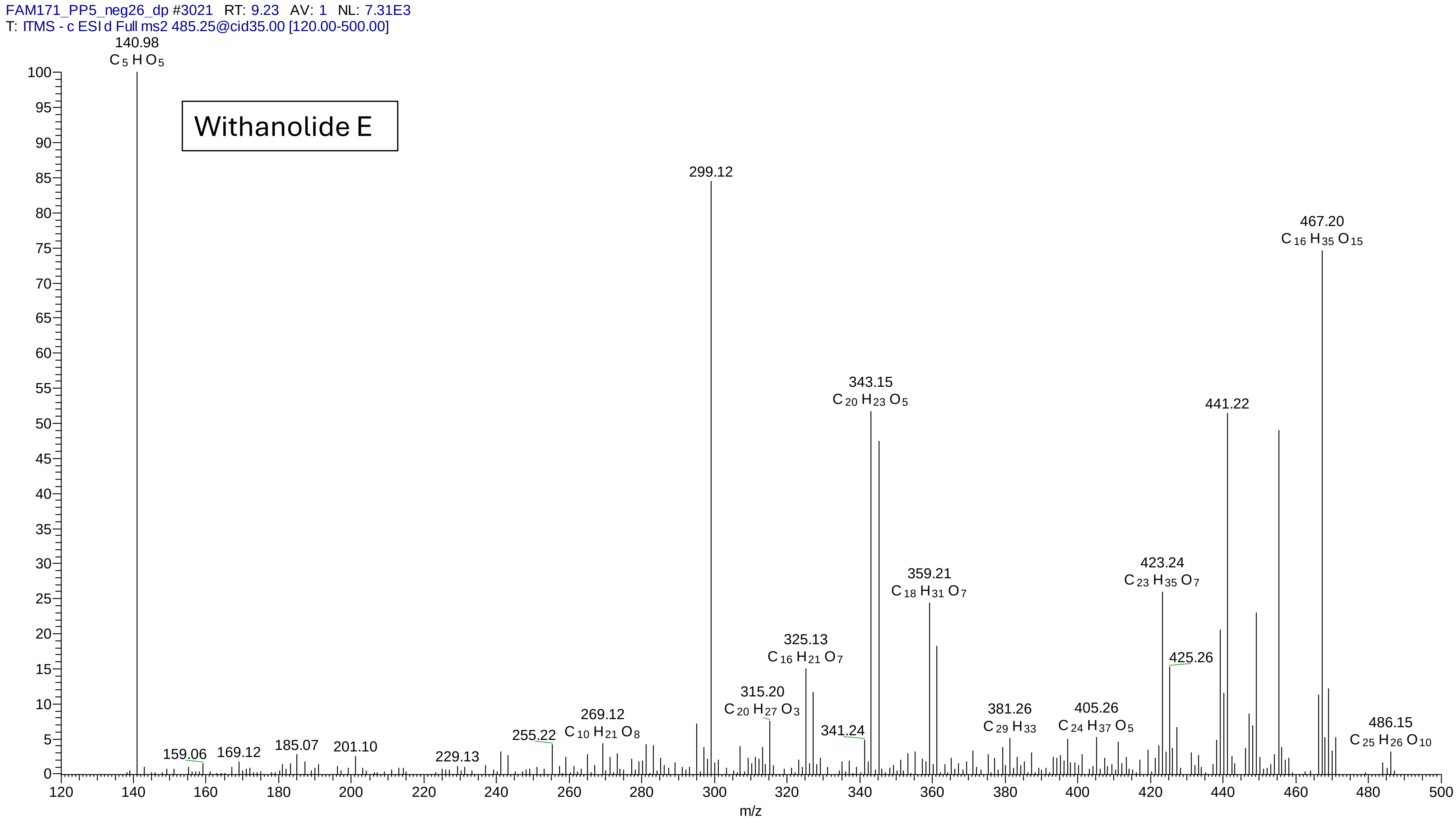

Fig.W1: MS/MS fragmentation spectrum of peak W6

Withanolide D

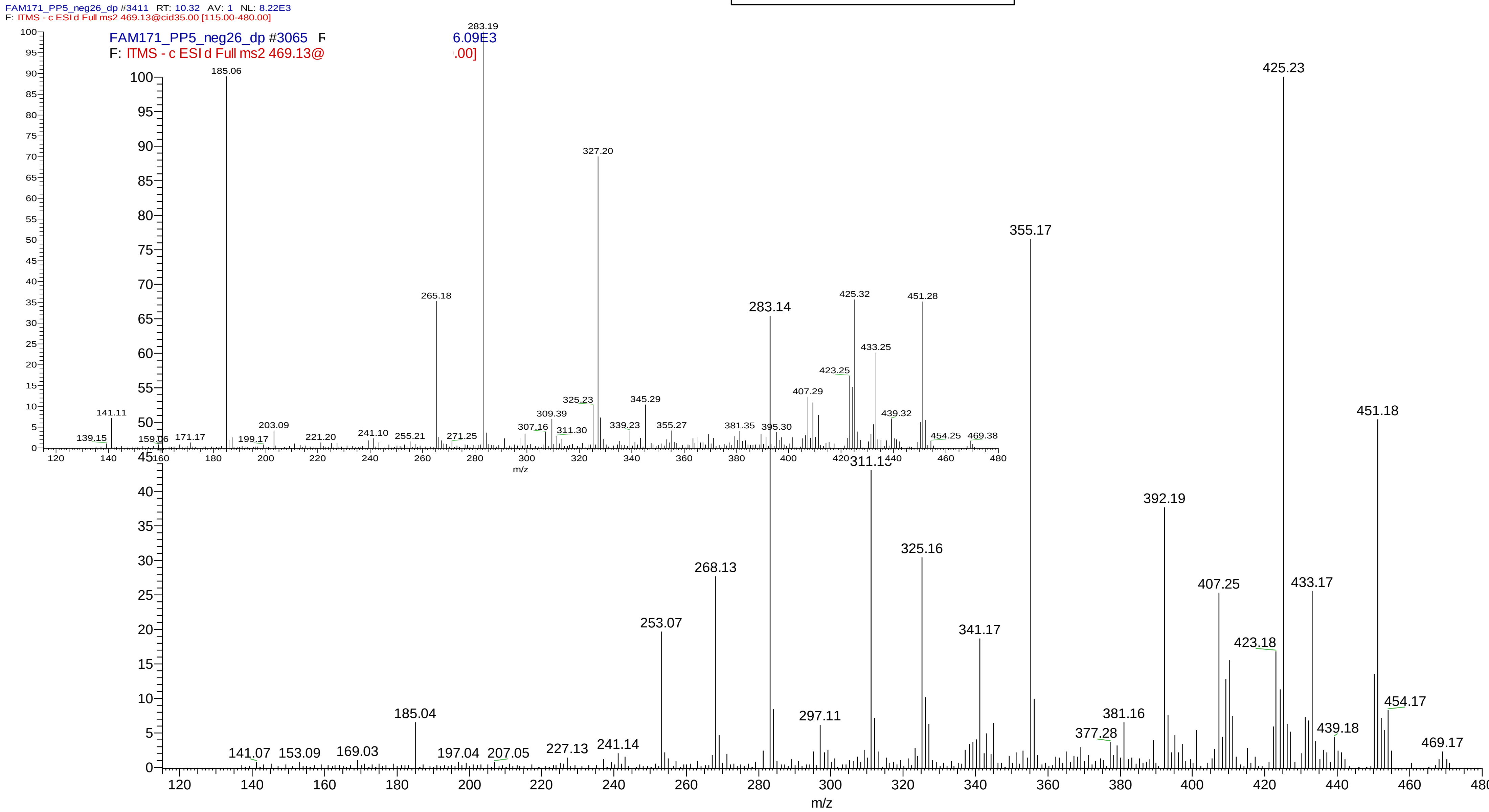

Fig.W2: MS/MS fragmentation spectrum of peak W7

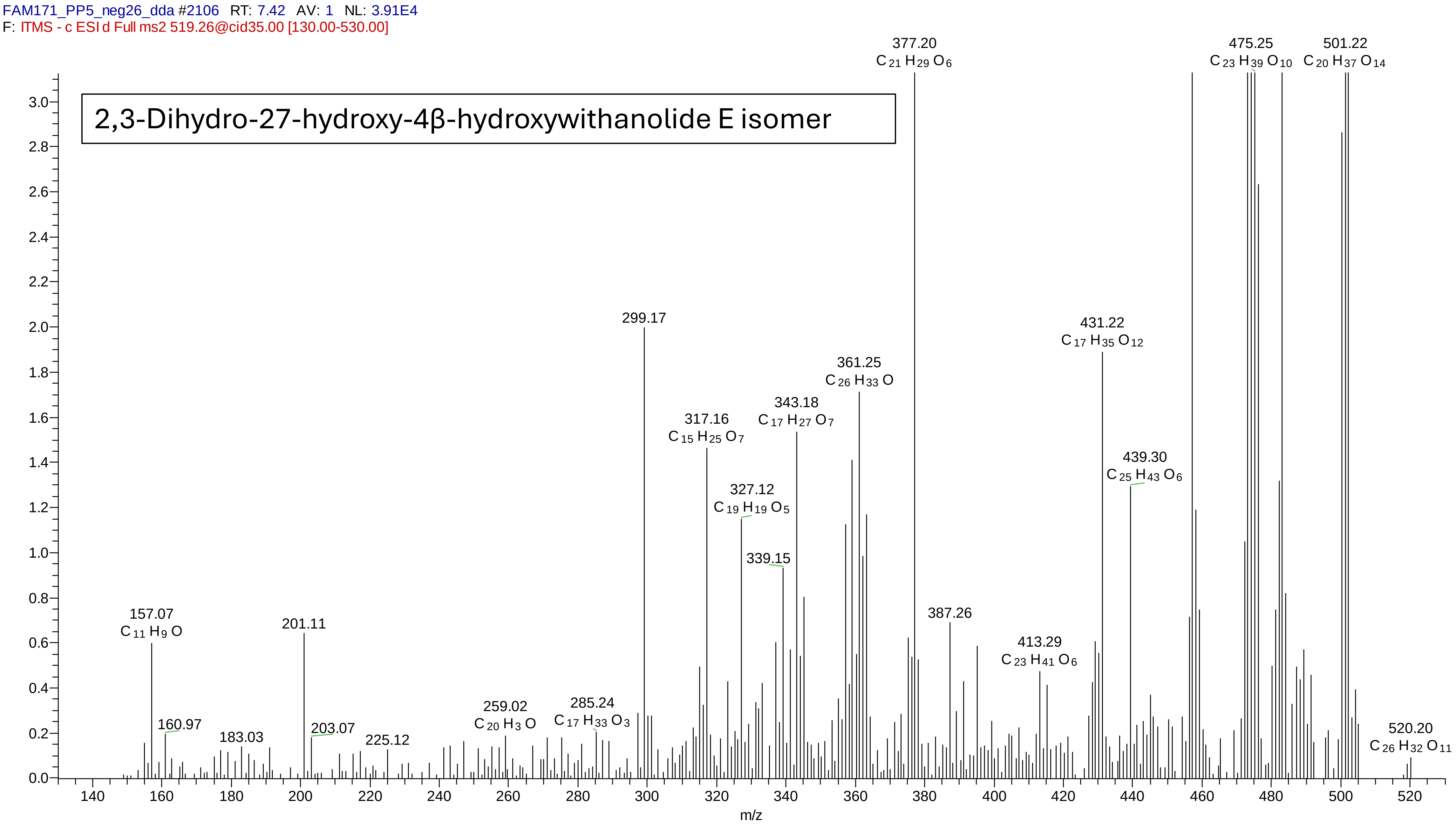

Fig.W3: MS/MS fragmentation spectrum of peak W2

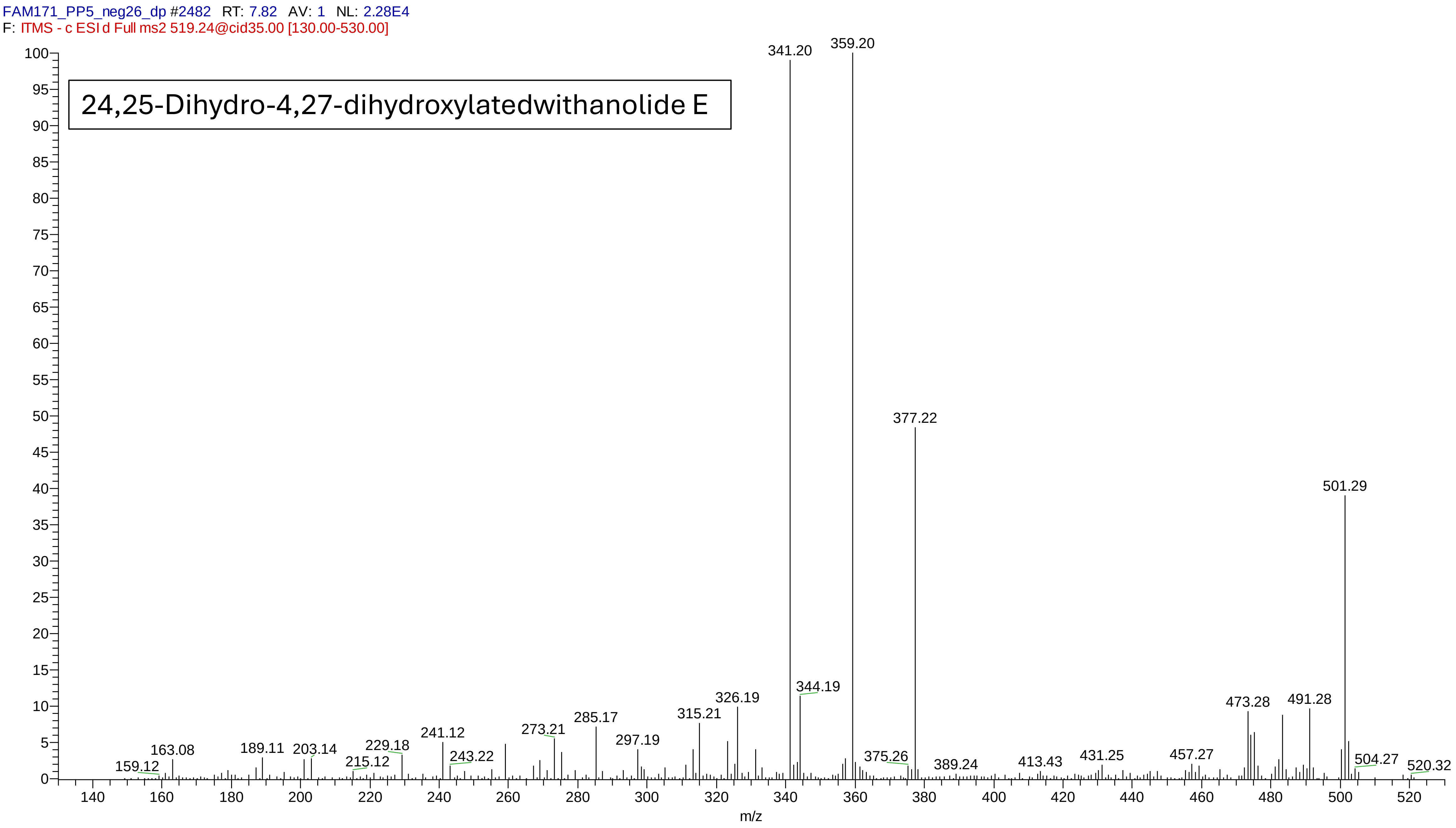

Fig.W4: MS/MS fragmentation spectrum of peak W4

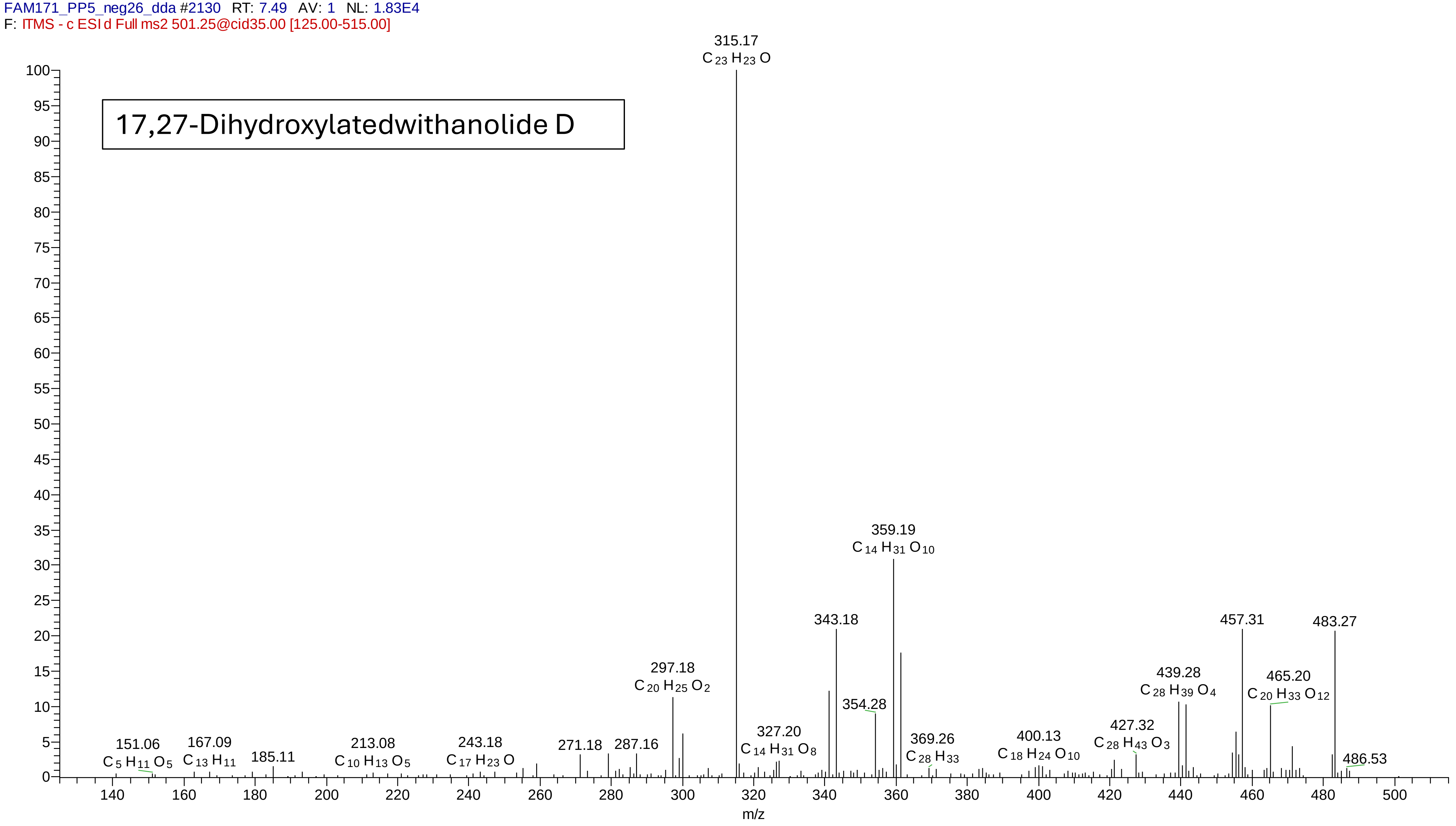

Fig.W5: MS/MS fragmentation spectrum of peak W3

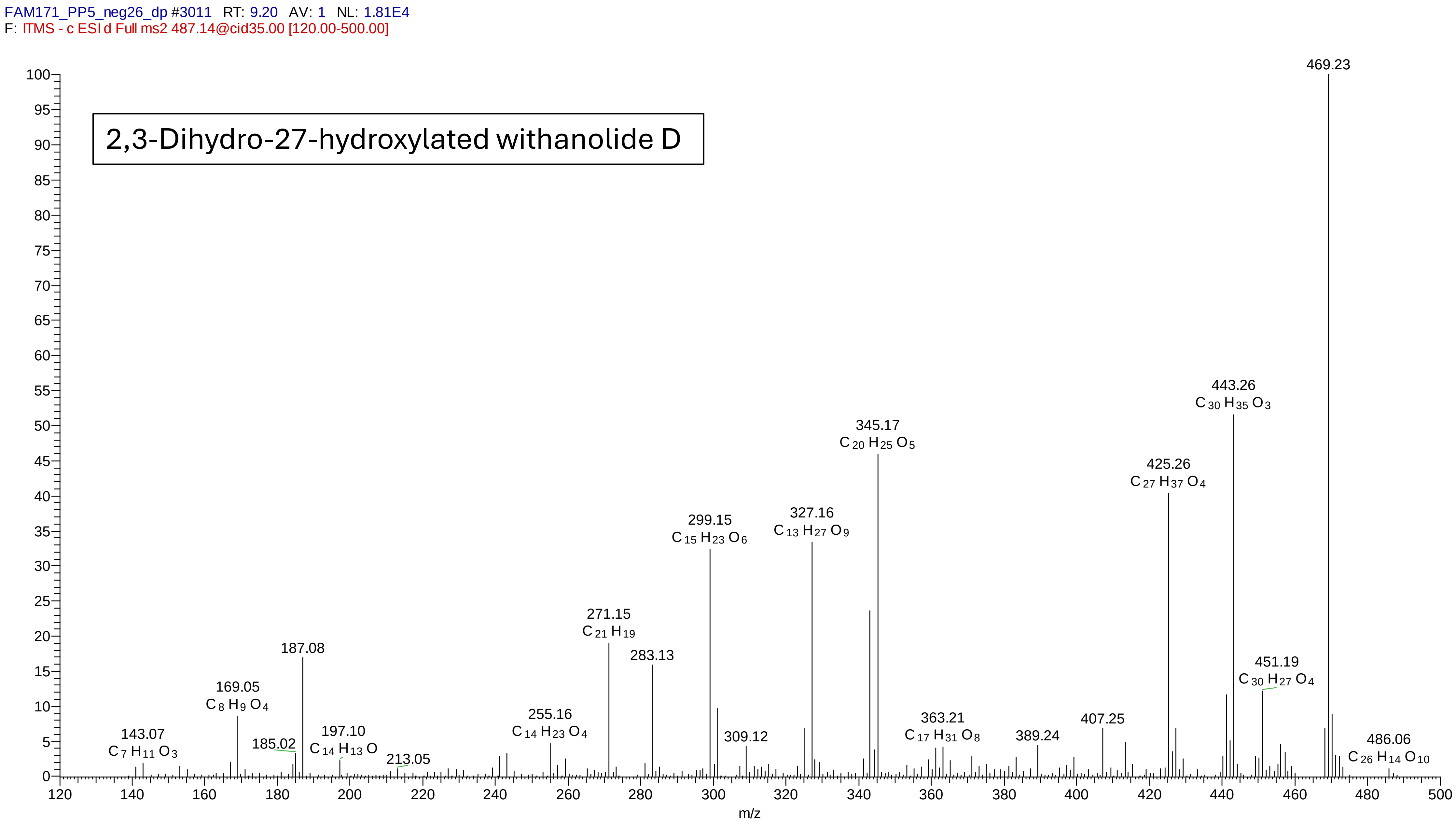

Fig.W6: MS/MS fragmentation spectrum of peak W5

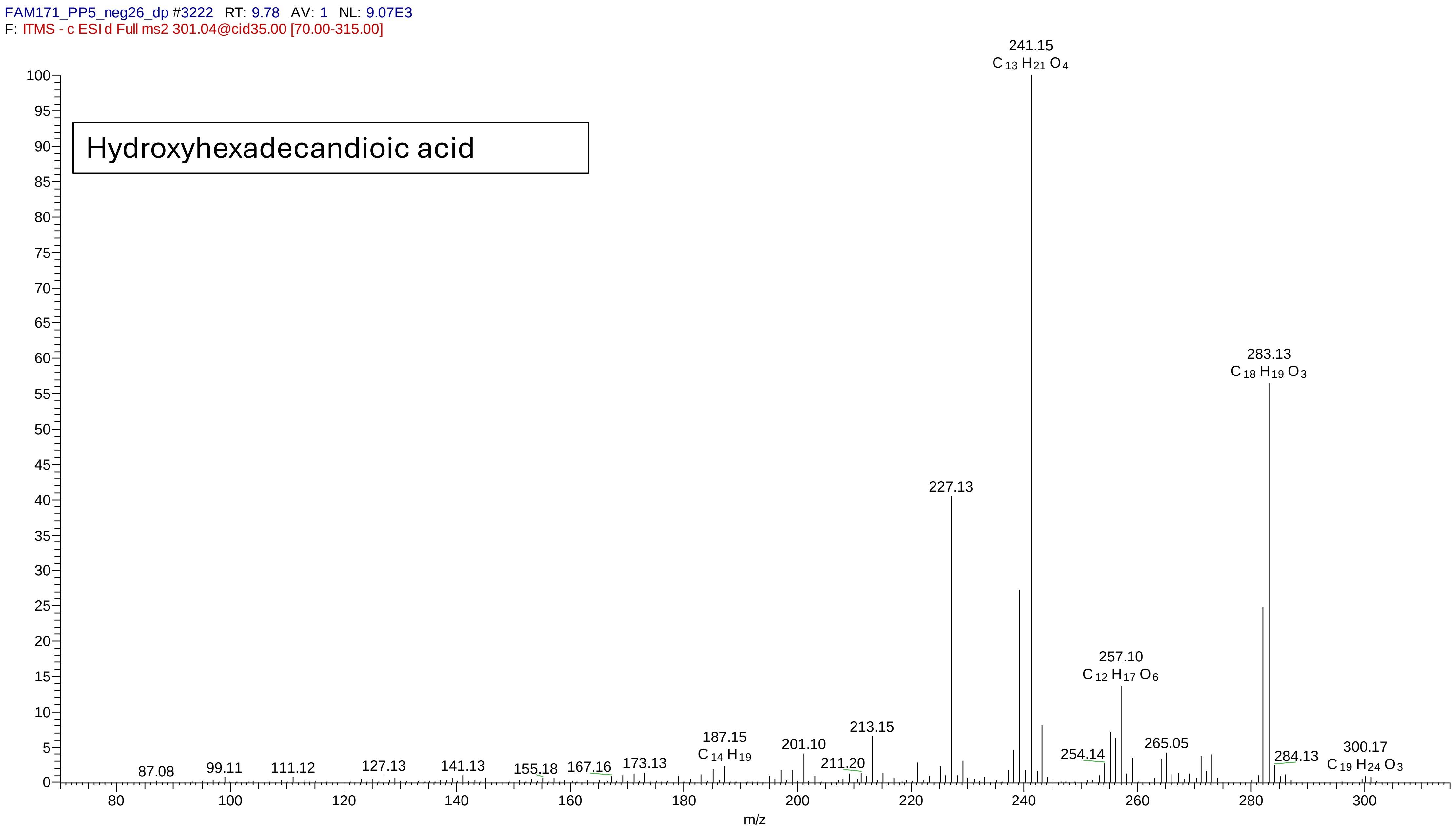

Fig.A1: MS/MS fragmentation spectrum of peak A1

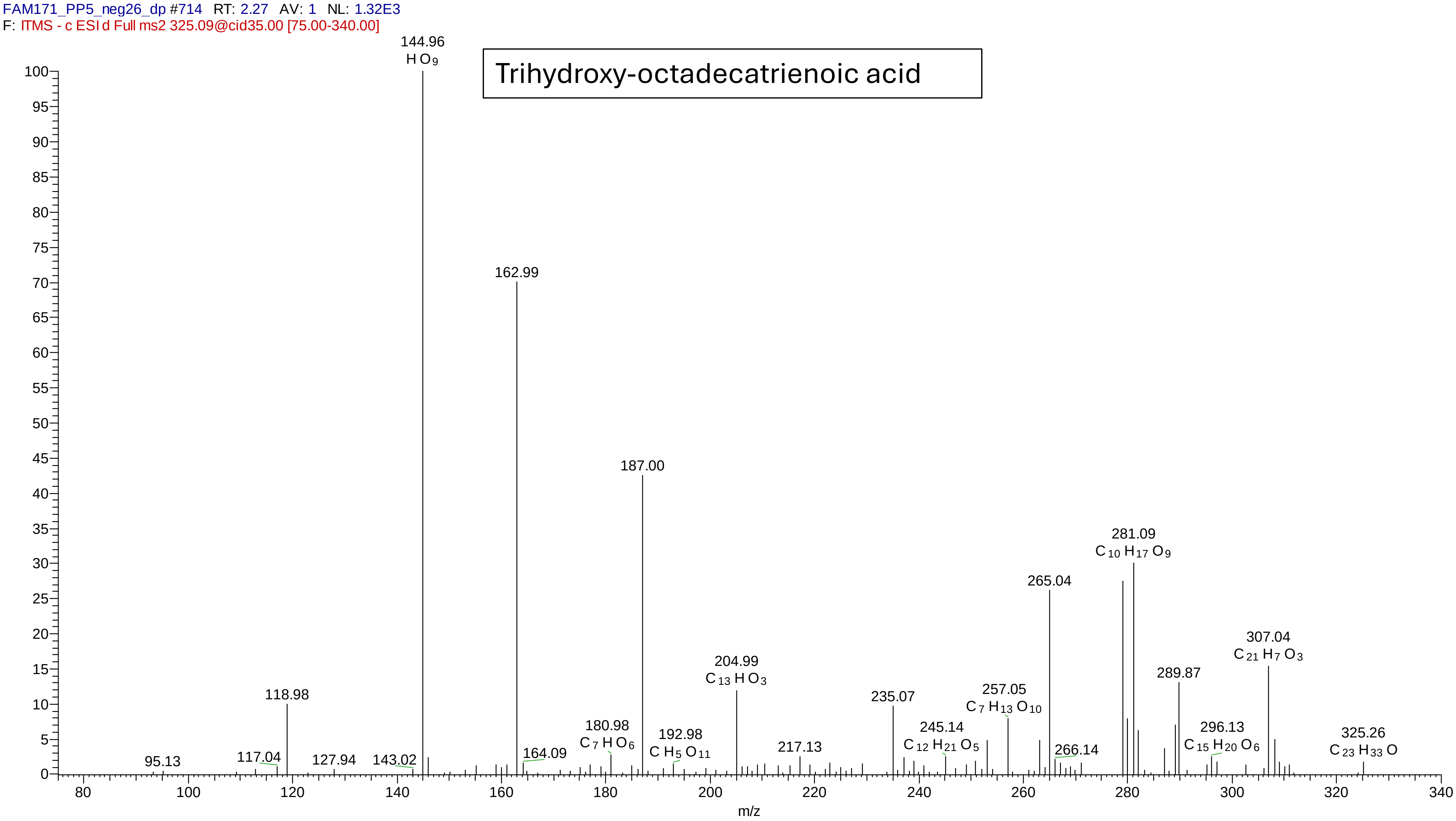

Fig.A4: MS/MS fragmentation spectrum of peak A2

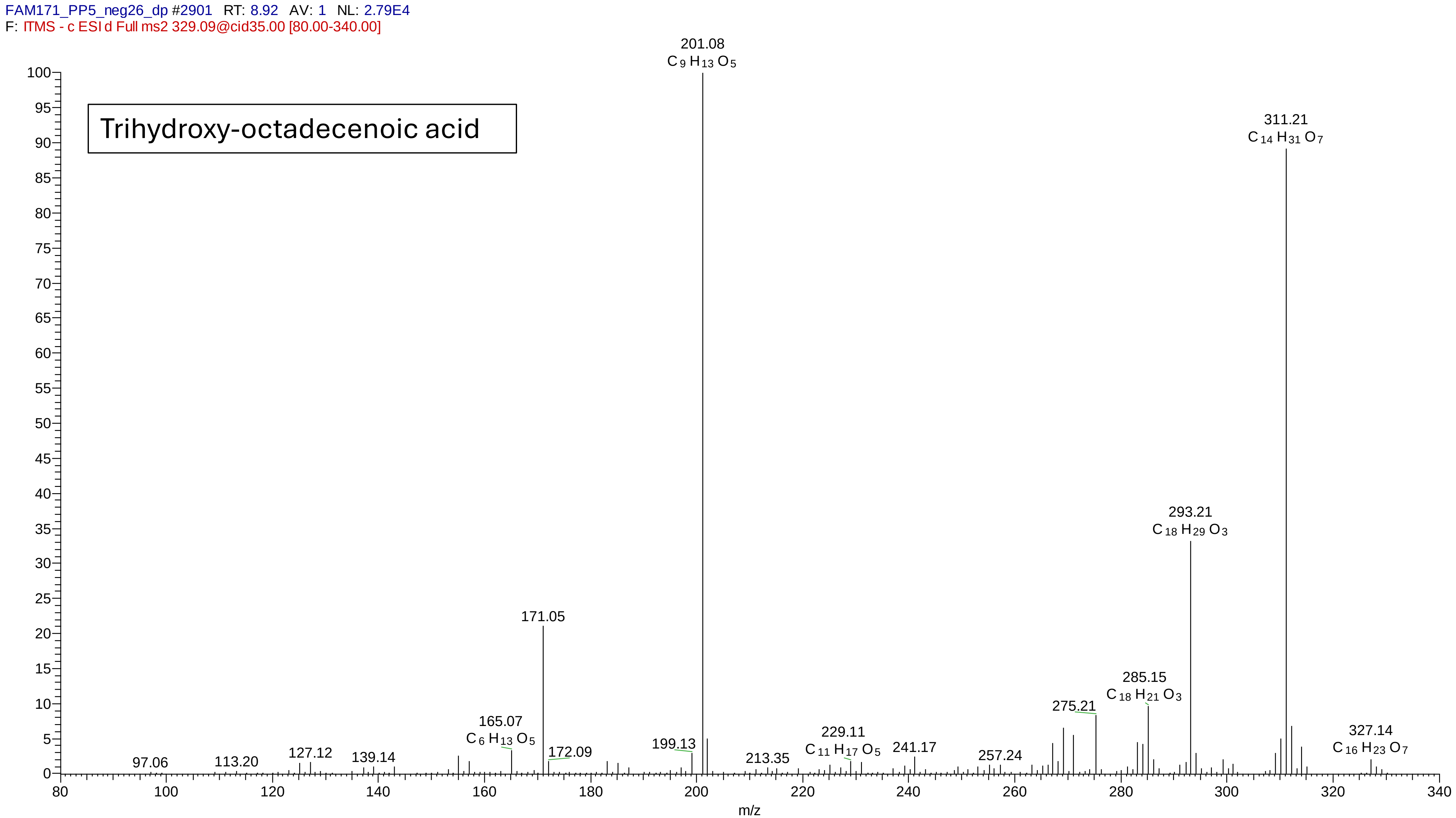

Fig.A3: MS/MS fragmentation spectrum of peak A3

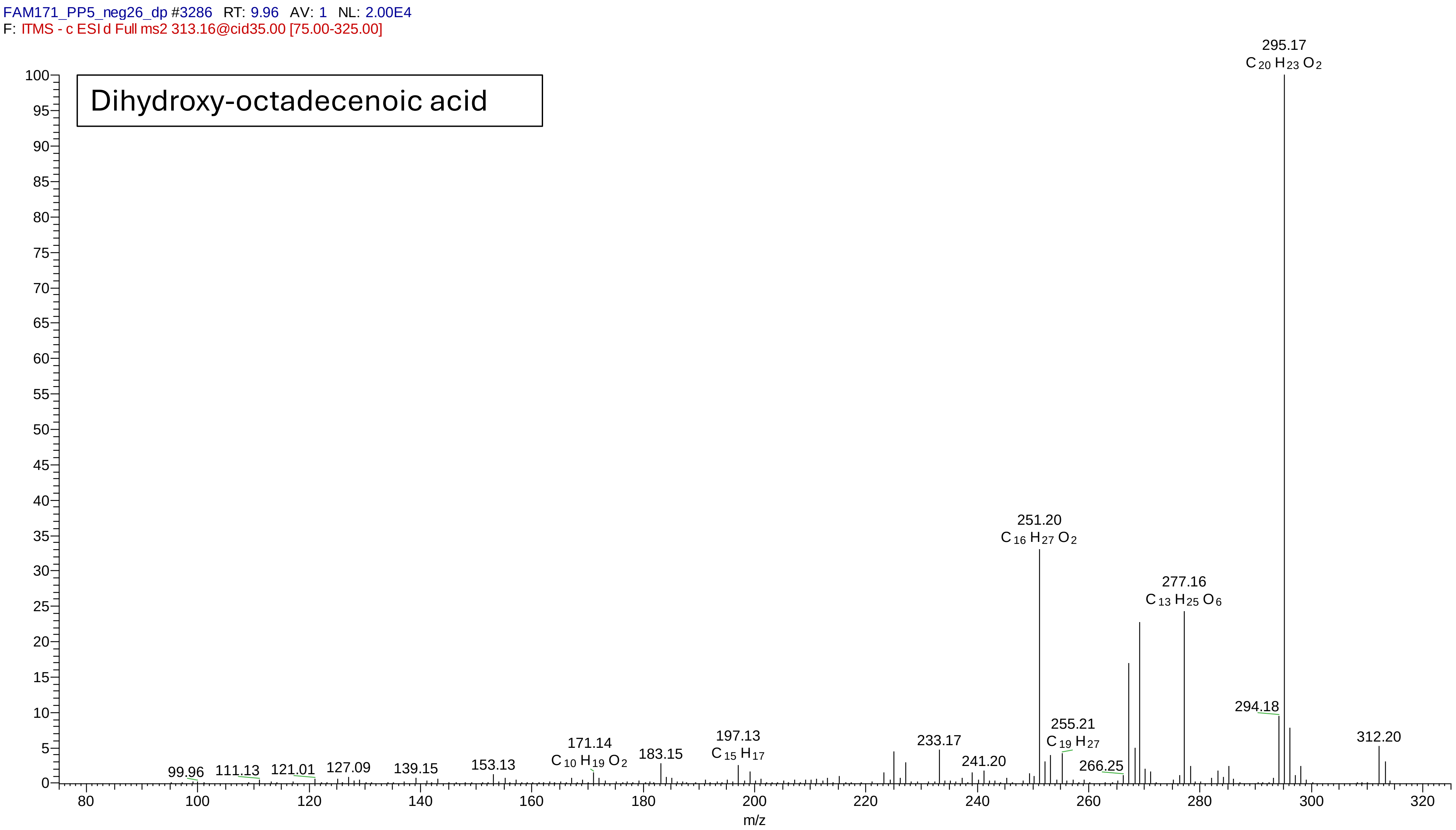

Fig.A2: MS/MS fragmentation spectrum of peak A4
